# Enhancing GABAergic Transmission Improves Locomotion in a *Caenorhabditis elegans* Model of Spinal Muscular Atrophy

**DOI:** 10.1523/ENEURO.0289-18.2018

**Published:** 2019-01-02

**Authors:** Chia-Yen Wu, David A. Gagnon, Juliette S. Sardin, Urva Barot, Alex Telenson, Paulo E. Arratia, Robert G. Kalb

**Affiliations:** 1 Department of Pediatrics, Division of Neurology, Research Institute, Children’s Hospital of Philadelphia, Philadelphia, PA 19104; 2 Department of Physics, Georgetown University, Washington, DC 20057; 3 Institute for Soft Matter Synthesis and Metrology, Georgetown University, Washington, DC 20057; 4 Department of Mechanical Engineering and Applied Mechanics, University of Pennsylvania, Philadelphia, PA 19104; 5 Department of Neurology, Perelman School of Medicine, University of Pennsylvania, Philadelphia, PA 19104

**Keywords:** aging, FOXO transcription factor, neuromuscular junction, SMN protein, suppressor

## Abstract

Spinal muscular atrophy (SMA) is a neuromuscular disease characterized by degeneration of spinal motor neurons resulting in variable degrees of muscular wasting and weakness. It is caused by a loss-of-function mutation in the survival motor neuron (*SMN1*) gene. *Caenorhabditis elegans* mutants lacking *SMN* recapitulate several aspects of the disease including impaired movement and shorted life span. We examined whether genes previously implicated in life span extension conferred benefits to *C. elegans* lacking SMN. We find that reducing daf-2/insulin receptor signaling activity promotes survival and improves locomotor behavior in this *C. elegans* model of SMA. The locomotor dysfunction in *C. elegans* lacking *SMN* correlated with structural and functional abnormalities in GABAergic neuromuscular junctions (NMJs). Moreover, we demonstrated that reduction in *daf-2* signaling reversed these abnormalities. Remarkably, enhancing GABAergic neurotransmission alone was able to correct the locomotor dysfunction. Our work indicated that an imbalance of excitatory/inhibitory activity within motor circuits and underlies motor system dysfunction in this SMA model. Interventions aimed at restoring the balance of excitatory/inhibitory activity in motor circuits could be of benefit to individuals with SMA.

## Significance Statement

Spinal muscular atrophy (SMA) is a pediatric motor neuron disease resulting from the loss of the survival motor neuron (SMN) protein. While great effort has been expended on interventions aimed at increasing levels of compensatory SMN1, identification of genes that modify the SMA phenotype has lagged. Here, we undertook a targeted genetic screen to identify SMA disease suppressors. We demonstrated that reduced insulin/insulin-like signaling is beneficial for not only longevity but also locomotor activity in SMA worm models. Our results from anatomic, functional, and genetic studies show that the impairment of GABAergic neurotransmission contributes to locomotor dysfunction in *smn-1* null worms. Enhancing GABAergic neurotransmission alone can correct the locomotor dysfunction. This work leads to new understanding of disease pathogenesis and opens up new opportunities for therapy.

## Introduction

Spinal muscular atrophy (SMA) is an autosomal recessive disease in infants and children, which is characterized by progressive degeneration of the motor neurons resulting from a reduction in abundance of functional survival motor neuron (*SMN*) protein (Lefebvre et al., 1997). SMN cycles between the nucleus and cytoplasm, and plays a critical role in assembly of the ribonucleoproteins (RNPs) required for pre-mRNA splicing (Fischer et al., 1997). In neurons, SMN is also found in axons and is thought to be important for RNA transport (Rossoll et al., 2003). Although SMN is ubiquitously expressed in all cells, clinically, the neuromuscular system displays the most prominent pathology in SMA. The cell type-specific pathology of disease is one of the more enigmatic aspects of SMA, it may reflect relative differences in tissue requirements for SMN-mediated biological processes (Monani, 2005; Zhang et al., 2008).

A variety of SMA animal models have been established including mice, zebrafish, flies, and worms. These platforms have provided insight into a number of critical pathophysiological questions (McWhorter et al., 2003; Oprea et al., 2008; Hua et al., 2011; Imlach et al., 2012). First, in which cell or cell types is SMN required for health and maintenance of the neuromuscular system? In mice, cell type-specific knock-out (KO) or cell type-specific rescue in an SMN deficient background suggests that the SMA phenotype is a result of loss of SMN from motor neurons (Martinez et al., 2012; Zhou et al., 2016), other neurons (Paez-Colasante et al., 2013), and muscle cells (Cifuentes-Diaz et al., 2001). In addition, peripheral tissues could be critical players, too (Hua et al., 2011). Second, is SMN required continuously to maintain neuromuscular system integrity or is there a critical window? Phenotypes of the conditional KO mice indicate that the period of neuromuscular junction (NMJ) formation/maturation (especially between postnatal day 10 and postnatal day 20) has a particularly high requirement for SMN, and adult animals have a substantially less, but non-trivial, requirement for SMN (Swoboda, 2011; Kariya et al., 2014). Third, which biological activities subserved by SMN are necessary for proper functioning of neuron? This has been challenging to unravel, but defects in splicing specificity are thought to be the main disease drivers (Zhang et al., 2008).

Humans have two copies of the *SMN* gene (*SMN1* and *SMN2*) that differ in a C/T nucleotide inside exon 7, which alters its splicing patterns. This critical difference results in an inclusion of exon 7 in the majority of *SMN1-*derived mRNA but an exclusion of exon 7 in the most of *SMN2*-derived mRNA. SMN protein lacking sequences encoded by exon 7 is un stable (Burnett et al., 2009). SMA is caused by the low levels of SMN protein, which results from loss of *SMN1* and variable compensation by the unstable products of the *SMN2* locus (Monani et al., 2000; Le et al., 2005). These observations on the molecular pathophysiology of SMN have therapeutic implications. In a mouse model of SMA, application of antisense oligonucleotide (ASO) therapy aimed at increasing the expression of exon 7-containing SMN from the *SMN2* locus have a major impact on disease (Hua et al., 2011), and human trials using this approach are promising (Finkel et al., 2016; Mercuri et al., 2018). Nonetheless, this therapeutic approach is problematic because ASOs do not cross the blood brain barrier, and intrathecal administration of ASO to fragile patients is a clinical issue. Non-SMN modifiers of the SMA phenotype have been identified (i.e., plastin 3 and ROCK). Targeting these molecules and the cell biological processes they impact has therapeutic potential (Oprea et al., 2008).

In an effort to identify new disease modifiers we asked whether genes previously implicated in life span extension of *Caenorhabditis elegans* (Kenyon, 2010) would influence the lifespan and locomotor phenotype of *smn-1* null animals. We discover reduction of the insulin/insulin-IGF signaling (IIS) pathway significantly extends lifespan and improves locomotor phenotypes in this SMA model. These effects are intriguing because reducing other genes previously implicated in lifespan extension did not extend lifespan in *smn-1* null animals. Furthermore, we further demonstrate that by loss of *daf-2*, locomotion is improved due to enhancement of GABAergic transmission. Our data suggest that impaired GABAergic transmission is disrupted normal homeostatic locomotion in SMA disease model. Interventions aims at restoring the balance of excitatory/inhibitor activity in motor circuits could be benefits to individuals with SMA.

## Materials and Methods

### *C. elegans* strains and handling

Worms were maintained at 20°C on Nematode growth media (NGM) plates seeded with *Escherichia coli* OP50 (Brenner, 1974). Stains used for this study are CB1370 *daf-2(e1370)III*, LM99 *smn-1(ok355) I/ht2 [bli-4(e937) qIs48] (I;III),* RM2710 *snf-11(ok156)* V, TJ356 *zIs356 IV [daf-16::daf-16a/b::GFP + rol-6]*, FY297 (also known as EG1653) *oxls22 [UNC-49B::GFP] II*, CZ333 *juIs1 [Punc-25-SNB_1_::GFP] IV*. Bruce Bamber and Anne Hart kindly shared FY297 *oxls22[UNC-49B-GFP]* and *smn-1(rt248),* respectively. N2 Bristol was used as the wild-type reference strain. Double or triple worms were generated by standard genetic crosses and verified by PCR or fluorescence expression (Table 1).

**Table 1.** A list of the *C. elegans* strains generated in this study and their corresponding genotype

Strain name	Genotype
RK110	*smn-1(ok355)/hT2;daf-2(e1370)*
RK115	*smn-1(ok355)/hT2;daf-2(e1370);juls-1 IV*
RK116	*smn-1(ok355)/hT2;daf-2(e1370);oxls22 II*
RK117	*smn-1(ok355)/hT2;juls-1 IV*
RK118	*smn-1(ok355)/hT2;oxls22 II*
RK120	*smn-1(ok355)/hT2;TJ356 [daf-16p::daf-16a/b::GFP + rol-6]*
RK122	*smn-1(rt248)/hT2;daf-2(e1370)*
RK123	*smn-1(ok355)/hT2;snf-11(ok156)*

### Lifespan analysis

Strains were grown at 20°C for at least two generations without starvation before lifespan analysis. Eggs were collected by bleaching reproductive adults of the desired genotype and letting them hatch and grow at either 20°C or 25°C. Survivors were monitored and counted every day. In all cases, *p* values were calculated using the Log-rank (Mantel–Cox) method.

### RNAi knock-down and survival assay in *C. elegans*

Each RNAi (RNA Interference) colony was grown overnight in Luria broth (LB) containing ampicillin (50 µg/ml), and 200 µl was seeded onto NGM plates containing isopropylthiogalactoside (IPTG; 1 mM) to induce dsRNA expression at room temperature. All RNAi clones used in this study were generated as described previously in the Ahringer library (Fraser et al., 2000) and were confirmed by DNA sequencing. Longevity-promoting RNAi candidates were validated in wild-type N2 animals. For lifespan experiments, ten young adult animals were placed on RNAi plates, eggs were laid for 4–6 h before hermaphrodites were removed. The progeny was allowed to grow on the RNAi plate until young adults (first generation). This cycle was repeated once more to obtain the third-generation progeny for the lifespan assay. Survivors were counted every day. An empty vector (EV) for RNAi constructs served as a control.

### Confocal images and synapse marker (puncta) analysis

Animals at L2 or Late (L2 + 3 d) stage were mounted on dried 2% agarose pads with M9 buffer. A stack of fluorescent images was captured from same posterior region of the dorsal nerve cord using Olympus laser scanning confocal microscopy with 100× objective lens. Stacked images were projected into a single plane using Fluoview software. The acquired images were processed and analyzed by an in house-developed PunctaAnalyzer program and R script (Hung et al., 2007).

### Pyridostigmine bromide and levamisole sensitivity assay

For analysis of sensitivity to the drugs pyridostigmine bromide and levamisole, three replicates of at least 20 worms per genotype were placed on NGM plates supplemented with either 100 mM pyridostigmine bromide (Sigma) or 100 µM levamisole (Sigma). Number of worms paralyzed on each plate was counted every 30 min to a total 360-min duration. Paralyzed worms were identified as those failing to response after 5 s of stimulation by plate-tapping and tail-prodding. The percentage paralyzed was averaged at each time point and plotted graphically using Log-rank (Mantel–Cox test) in PRISM.

### Quantitative RT-PCR analysis

We hand-picked 500 worms of the various genotypes and at the examined ages for extraction of total RNA using TRIzol (Invitrogen) and RNeasy Mini kit (QIAGEN). A total 1 µg of DNase-treated RNA were reverse transcribed using iScript Reverse Transcription Supermix (Bio-Rad) according to the manufacture’s protocol. The cDNAs were subjected to real-time qPCR in a total volume of 25 µl, containing 1× Power SYBR Green PCR Master mix (Applied Biosystems) and 200 nM primer. The qPCRs were amplified and analyzed in triplicate using StepOne Real-Time PCR system (Invitrogen). Relative mRNA level of the genes of interest were normalized against α tubulin (*tba-1*) as an endogenous control. Primer sequences for amplification of target mRNA were as following: *smn-1*, 5′-ATACCTCGATGCCATTTCCA-3′ and 5′-ATCCGCTCATGTACCAGCTC-3′; *tba-1*, 5′-AAGATGCCGCCAACAACTAC-3′ and 5′-CCTCCTCCGAATGAATGAAA-3′.

### Swimming assay and biomechanical profile analysis

We captured movies of swimming nematodes in sealed fluidic chambers that are 2 cm in diameter and 1 mm in depth. Imaging is performed using bright-field microscopy and a high-resolution CCD camera at 30 frames per second. We discard all movies that exhibit nematode-wall interactions and interrupted or irregular beating, as well as movies with fewer than four (worm) beating cycles. Excluding discarded movies, we obtain an average of 20 movies of unique individuals per group tested, with a minimum of 11.

Movies of swimming nematodes are analyzed using in-house software with slight modification from the published software which was previously used and validated for a study on biomechanical profiling of *C. elegans* genotypes (Krajacic et al., 2012). The original software is freely available as supplemental material from the *Genetics* journal website (http://www.genetics.org/content/191/3/1015.supplemental). The modified version of software in this study is freely available via the University of Pennsylvania Complex Fluids Lab website (http://arratia.seas.upenn.edu/software.html). The modifications allow the code to be used for the study of immature rather than adult *C. elegans*. We have implemented changes that allow us to accurately measure the biomechanical properties of younger and therefore smaller and thinner worms by replacing parameters valid for adult, approximately millimeter-length *C. elegans* with parameters calculated directly from measured lengths and diameters.

The software uses image processing techniques to track the swimming motion of *C. elegans* by extracting the body-shape of the swimmer and then computing the nematode's biomechanical properties, including frequency, amplitude, speed, force, and power (Krajacic et al., 2012). In brief, the nematode’s body is separated from the background of the image, and its centroid and body shape in each frame in the plane of motion is computed as well as the locations of its head and tail. Differentiating the centroid position in time yields the forward swimming speed U. Next, we divide the centerlines into segments, which are tracked in space and time to create a parameterized body shape s(x,y,t), where x and y define the plane of motion and t represents time. The nematode’s bending curvature κ is defined as the change in local orientation along the body of the worm. When plotting curvature as a function of time, diagonal stripes of alternating positive and negative curvature clearly show the periodicity of the nematode’s swimming gait, where the beating frequency is defined as f=1/T, where T represents the time to complete one beating cycle.

We also calculate the normalized curvature as a function of position along the nematode by first averaging the local curvature in time to obtain $\overset{-}{\kappa }(s)$ for each individual nematode. We then take the magnitude, or absolute value, of $\overset{-}{\kappa }(s)$, the average across each population, and normalize by the average curvature at the head of the worm to compare across genotypes. Note that we neglect the first and last 5% of the nematode’s parameterized body shape due to its decreasing diameter and increasing transparency which introduce uncertainties in our curvature measurements.

Moreover, we use the measured body shapes and velocities to compute factors that might differentiate the behavior of nematodes with SMA from healthy wild-type organisms. We use a simple hydrodynamic model, resistive force theory (Gray and Hancock, 1955), to estimate the propulsive force of the nematode (Sznitman et al., 2010). Since *C. elegans* are self-propelled and force-free (Lauga and Powers, 2009), the sum of its propulsive and drag forces must be zero such that

$$F_{P}+F_{D}=0,$$

where $F_{P}$ and $F_{D}$are the propulsive and drag forces acting on the nematode, respectively. We can then estimate $F_{P}$ by computing $F_{D}$ from RFT; for each body segment, we can decompose its force contribution as:

$$dF(t)=\left(F_{N}+F_{T}\right)ds=\left(C_{N}u_{N}+C_{T}u_{T}\right)ds,$$

where $F_{N}$ and $F_{T}$ are the normal and tangential components of the drag force, $C_{N}$ and $C_{T}$ are the normal and tangential components of the drag coefficient, and $u_{N}$ and $u_{T}$ are the normal and tangential components of the velocity of each body segment, respectively. The drag coefficients $C_{N}$ and $C_{T}$ are given by Lighthill’s refinement of the original RFT coefficients (Gray and Hancock, 1955; Lighthill, 1976). Performing the integral over the surface of the worm, we can estimate propulsive force (Lauga and Powers, 2009; Sznitman et al., 2010) using:

$$F_{P}(t)=\int_{S}^{}\left(C_{N}u_{N}+C_{T}u_{T}\right)ds.$$

The calculation of period-averaged propulsive force provides an estimate of muscle (mechanical) output, and more intuitively, as an indirect measure of strength.

Furthermore, we can compute the power, or rate of energy expenditure, of *C. elegans* by multiplying the component of the propulsive (or drag) forces for each segment by its local velocity components:

$$P\left(t\right)=\int_{S}^{}\left(F\cdot u\right)ds=\int_{S}^{}\left(C_{N}{u_{N}}^{2}+C_{T}{u_{T}}^{2}\right)ds.$$

A period-averaged calculation of power is therefore an estimate for how much mechanical energy is consumed by *C. elegans* for locomotion.

### Statistics

Statistical analysis was performed using GraphPad Prism. For data containing more than three groups of non-normally distributed data, a non-parametric Kruskal–Wallis test with Dunnett’s multiple comparisons test was used. For normally distributed data (puncta analysis), one-way ANOVA with the *post hoc* Tukey HSD was applied for multiple comparisons. For survival analysis, a Log-rank (Mantel–Cox) test was used. The threshold for significance was set to *p <* 0.05. For normalized body curvature, multiple *t* tests followed by Holm–Sidak method correction were used.

## Results

### Inactive daf-2/IGF-1-like signaling pathway extend lifespan in SMA worm models

To identify new SMA disease modifier, we studied a *C. elegans smn-1* null mutant *ok355* (gene name C41G7.1). The null allele is maintained in a heterozygous state using the hT2g balancer chromosome and heterozygous animals (*smn-1(ok355)/*hT2g) are phenotypically normal. Maternally contributed SMN message to the developing embryo allows for the *smn-1(ok355)* mutant animals to develop and mature until their premature demise before adulthood. Animals homozygous for the null allele *smn-1*(*ok355*) have two prominent phenotypes: (1) impaired locomotion (e.g., uncoordinated or “unc”), and (2) death at 7–8 d of life at 20°C (Briese et al., 2009). Here, we focused on these phenotypes due to their potential relevance to the SMA disease.

In *C. elegans*, both lifespan and stress resistance can be increased by the loss of function (LOF) of a variety of single genes that influence caloric intake (Houthoofd et al., 2002), mitochondrial respiration (Dillin et al., 2002), insulin/IGF-1-like signaling (IIS; Kimura et al., 1997), or germline function (Arantes-Oliveira et al., 2002). We hypothesized that such genes and/or its downstream targets could be SMA disease modifiers. Thus, we took a RNAi screen approach and asked whether any of these longevity-promoting genes conferred a survival benefit for *smn-1(ok355)* mutant animals by RNAi-mediated knock-down. Nine RNAi candidates were selected (Extended Data Fig. 1-1; Kenyon et al., 1993; Ogg et al., 1997; Jia et al., 2004; Hamilton et al., 2005; Hansen et al., 2005; Oh et al., 2006; Olsen et al., 2006; Shen et al., 2014). Surprisingly, only knock-down of the insulin/IGF-1-like receptor gene *daf-2* significantly extended the life-span of *smn-1(ok355)* mutant animals (Extended Data Fig. 1-1). Knock-down of *age-1* did not confer a benefit (despite its known relationship to *daf-2* signaling) and this might be due to compensation by the parallel insulin receptor substrate (IST-1) pathway (Wolkow et al., 2002).

**Figure 1.**
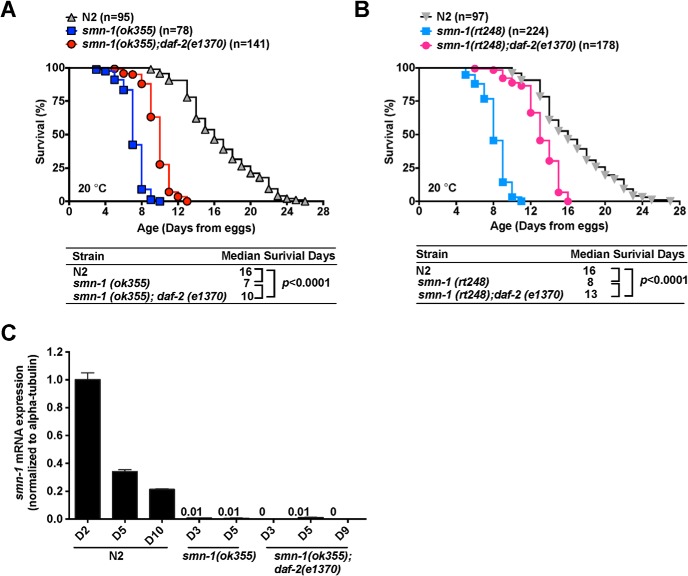
Inactivation of daf-2/IGF-1 like signaling pathway extend lifespan in SMA worm models. ***A***, Lifespan analysis of N2, *smn-1(ok355)*, and *smn-1(ok355);daf-2(e1370)* mutant animals at 20°C. *Smn-1(ok355);daf-2(e1370)* homozygotes live significantly 30% longer than *smn-1(ok355)* mutant animals (*p* < 0.0001, Log-rank test; for additional details, see Extended Data Fig. 1-1). ***B***, Lifespan analysis of N2, *smn-1(rt248)*, and *smn-1(rt248);daf-2(e1370)* at 20°C. *Smn-1(rt248);daf-2(e1370)* homozygotes live significantly 40% longer than *smn-1(ok355)* mutant animals (*p* < 0.0001, Log-rank test). ***C***, Quantitative analysis of *smn-1* mRNA in wild-type N2, *smn-1(ok355)*, and *smn-1(ok355);daf-2(e1370)* mutant animals at various developmental time points. qPCR (*n* = 3/timepoint/strain) measuring *smn-1* mRNA abundance normalized to *tba-1* in indicated strains is shown in the *y*-axis. Levels of *smn-1* mRNA in N2 animals gradually decrease from day 2 (D2) to day 10 (D10) after hatch. Levels of *smn-1* mRNA in both *smn-1(ok355)* and *smn-*1(*ok355);daf-2(e1370)* mutant animals were barely detectable (<1.0% of *smn-1* mRNA in N2). The bar graph represents mean ± SEM from three independent experiments.

To verify the survival benefit of *smn-1(ok355)* due to down-regulated *daf-2*, we placed the *smn*-1 null allele in the *daf-2 (e1370)* background. The *e1370* allele is a temperature-sensitive, partial loss-of-function (LOF) allele of *daf-2*. At 20°C, *smn-1(ok355);daf-2(e1370)* double mutants exhibited a median survival time of 10 d, living significantly longer than *smn-1(ok355)*, which have a median survival time of 7 d (Fig. 1*A*; Log-rank test, *p* < 0.0001). Recently, a second described SMA worm model (*smn-1(rt248)*; Dimitriadi et al., 2016) is established, which also exhibited a shortened life span (median survival of 8 d). We generated the *smn-1(rt248);daf-2(e1370)* mutant animals and observed that they exhibited a significant prolongation survival (median survival 13 d; Fig. 1*B*; Log-rank test *p* < 0.0001). Taken together, our results suggest that loss of *daf-2* or *daf-2* inactivation can provide a survival benefit in worm models of SMA.

10.1523/ENEURO.0289-18.2018.f1-1 Extended Data Figure 1-1 An approach of RNAi screen identified Daf-2 as a disease modifier to promote lifespan in *smn-1(ok355)* mutant animals. ***A***, Lifespan analysis of wild-type N2 worms fed either EV (median survival 14 d) or corresponding RNAi clones, including *age-1* RNAi (median survival 18 d), *daf-2* RNAi (median survival 21.5 d, *p* < 0.001, Log-rank test), *daf-15* RNAi (median survival 14 d), C27B7.7 RNAi (median survival 25 d, *p* < 0.001, Log-rank test), *cdc25* RNAi (median survival 15 d, *p* < 0.001, Log-rank test), *ril-1* RNAi (median survival 29 d, *p* < 0.001, Log-rank test), *sca-1* RNAi (median survival 13 d), *yrs-1* RNAi (median survival 13 d), or *rha-1* RNAi (median survival 14 d); *p* < 0.005 was considered significant after Bonferroni correction. ***B***, Lifespan analysis of *smn-1(ok355)* mutant animals fed either EV (median survival 8 d) or longevity-promoting RNAi clones validated in Figure 1*A*. Only *daf-2* RNAi feeding significantly prolongs lifespan in *smn-1(ok355)* mutant animals (median survival 11 d, *p* < 0.0001, Log-rank test); *p* < 0.006 was considered significant after Bonferroni correction. ***C***, Table for statistical analysis of lifespan data in RNAi screen and reference of selected RNAi candidates. After conducting a literature search, nine candidate RNAi clones, which can extend lifespan in *C. elegans*, were selected for RNAi screen experiment. Download Figure 1-1, TIF file.

A critical function of SMN protein is the assembly of small nuclear RNPs (snRNPs), which are required for pre-mRNA splicing machinery (Gubitz et al., 2004). This suggests that *smn-1(ok355)* mutant animals may die prematurely when maternally derived SMN mRNA and protein levels fall below a specific threshold although this threshold varies as a function of developmental stage and in a cell type-specific manner. The striking beneficial effect of loss of *daf-2* on *smn-1(ok355)* mutant animals’ survival could arise from two possibilities. First, loss of *daf-2* might slow the degradation of *smn-1* mRNA or protein and thereby maintaining them above the critical threshold. Second, loss of *daf-2* might render animals resistant to reduced SMN protein levels.

To address these possibilities, we conducted real-time quantitative PCR to monitor *smn-1* RNA level in wild-type N2, *smn-1(ok355)*, and *smn-1(ok355);daf-2(e1370)* mutant animals at various developmental time points (Fig. 1*C*). In wild-type N2 animals, we found a progressive decline in the *SMN* messenger RNA abundance from day 2 to day 7 on hatching. Compared to *smn-1* RNA level from N2 animals, barely detectable *smn-1* RNA levels were found in both *smn-1(ok355)* and *smn-1(ok355);daf-2(e1370)* strains, across multiple developmental time points. This result suggests that loss of *daf-2* activity prolongs lifespan in *smn-1(ok355);daf-2(e1370)* strain despite the lack of *smn-1* mRNA. The absence of good tools for monitoring SMN protein levels leaves a caveat that loss of *daf-2* might slow SMN protein degradation. In mammalian cells, the half-life of full length SMN protein is ∼4.5 h (Burnett et al., 2009). It seems unlikely that animals lacking SMN mRNA for 5+ d after hatching would still have functional SMN protein. Thus, it suggests that loss of *daf-2* renders *smn-1(ok355)* mutant animals insensitive to the absence of SMN protein.

Taken together, our results reveal that not every longevity promoting pathways identified in *C. elegans* are capable to provide survival benefit in *smn-1* null animals. Only reduction of *daf-2* signaling pathway has such unique ability to extend the life of animals lacking SMN-1. The beneficial effects of reducing *daf-2* appear to be independent of changes in SMN mRNA and probably protein.

### Activation of Daf-16 is required for lifespan extension in *daf-2*-dependent pathway in *smn-1* null animals

**Figure 2.**
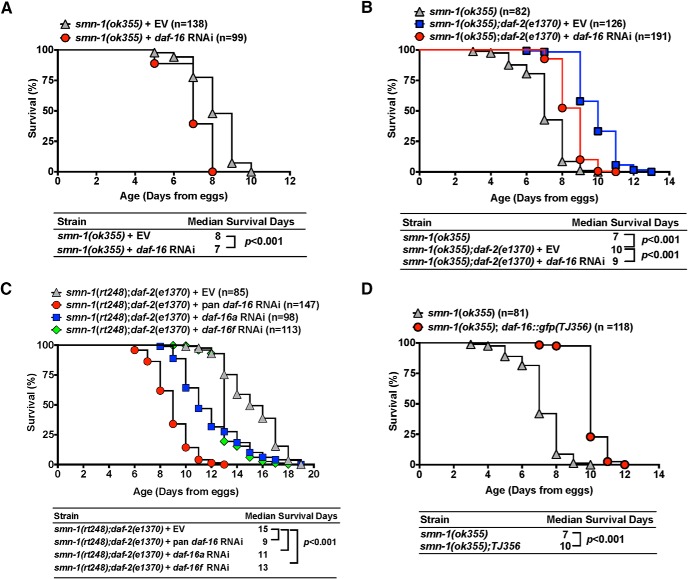
Enhanced survival benefits in SMA worm models are mediated by the *daf-2/daf-16* pathway. ***A***, Knock-down *daf-16* by RNAi feeding shortens lifespan of *smn-1(ok355)* mutant animals by 12.5% when compared to EV (*p* < 0.001, Log-rank test). ***B***, *smn-1(ok355);daf-2(e1370)* mutant animals live three more days than *smn-1(ok355)* mutant animals, and this beneficial effect is suppressed by feeding RNAi targeting *daf-16* (*p* < 0.001, Log-rank test). ***C***, *smn-1(rt248);daf-2(e1370)* mutant animals live 15 d, and this beneficial effect is suppressed by feeding RNAi that targets all isoforms of *daf-16* (*p* < 0.001, Log-rank test). Feeding RNAi that targets *daf16a* or *daf16f* isoforms also suppress the longevity of *smn-1(rt248);daf-2(e1370)* mutant animals although the effect is less robust than the pan *daf-16* RNAi effect (*p* < 0.001, Log-rank test). ***D***, Overexpression of *daf-16* using *TJ356 (daf-16::gfp)* strain prolongs lifespan of *smn-1(ok355)* mutant animals by 43.8% when compared to *smn-1(ok355)* mutant animals (*p* < 0.001, Log-rank test).

In wild-type worm, it has been shown that a reduction of *daf-2* signaling can double the lifespan (Lin et al., 1997; Ogg et al., 1997; Lee et al., 2001) and such extended lifespan phenomenon is dependent on its downstream target, DAF-16/FOXO transcription factor, which is negatively regulated by *daf-2* signaling (Larsen et al., 1995; Dillin et al., 2002). Thus, we examined whether the increased lifespan effect in *smn-1(ok355);daf-2(e1370)* is also daf-16/FOXO dependent. Both *smn-1* and *daf-16* reside on Chromosome I (about one centimorgan apart), and it is difficult to generate animals that contain mutations of both genes in this case. To examine whether DAF-16 is required and sufficient for lifespan extension in *smn-1* null worms, we used both RNAi and overexpression approaches. *smn-1(ok355*) mutant animals fed with *daf-16* RNAi showed 12.5% median lifespan decrease in comparison with EV-fed animals (Fig. 2*A*; *p* < 0.001, Log-rank test). Feeding *daf-16* RNAi to *smn-1(ok355);daf-2(e1370)* and *smn-1(rt248);daf-2(e1370)* mutant animals suppressed the longevity-promoting effect *by daf-2(e1370)* (Fig. 2*B*,*C*). The effect of reduced *daf-16* by feeding RNAi was incomplete, which may be ascribed to the incomplete knock-down of *daf-16* or *daf-16*-independent effects of *daf-2*. Several splicing-isoforms of DAF-16 exist and they mediate expression of overlapping and distinct gene sets (Chen et al., 2015). Thus, we asked whether a specific isoform of *daf-16* is involved in the lifespan effect. We found that feeding RNAi specifically targeting *daf-16a* or *daf-16f* (Chen et al., 2015), was less effective than pan *daf-16* feeding RNAi in suppressing the beneficial actions of *daf-2(e1370)* on *smn-1(ok355)* mutant animals’ longevity (Fig. 2*C*). This indicates that target genes regulated by both isoforms contribute to longevity of animals with reduced SMN abundance. To overexpress DAF-16 in *smn-1* null animals, we used the *TJ356(daf-16::gfp)* strain in which a 6 kB of *daf-16* promoter drives the expression of DAF-16-GFP fusion proteins (Henderson and Johnson, 2001). There is 42% median lifespan increase for *smn-1(ok355);daf-16::gfp(TJ356)* mutant animals comparison with *smn-1(ok355)* mutant animals (Fig. 2*D*; *p* < 0.001, Log-rank test). Our results suggest that *daf-16* is required for *daf-2*-dependent lifespan extension of *smn-1(ok355)* mutant animals.

### Reduced *daf-2* signaling improves the locomotor activity of *smn-1* mutant animals

Based on these observations with *daf-2* LOF we explored our main interest – the impaired locomotion of the *smn-1(ok355)* mutant animals. (Briese et al., 2009). Although *smn-1(ok355)* mutant animals display impaired locomotor activity, there is neither neuronal death nor gross anatomic abnormalities in motor circuit wiring (Briese et al., 2009). This suggests that functional deficits within the circuitry precede anatomic abnormalities, an observation commonly made in neurodegenerative disease models (Ilieva et al., 2008; Shakkottai et al., 2011). An alternative explanation is that relevant, but subtle anatomic defects require higher resolution examination. In the mouse SMA models, the circuitry devoted to controlling motor neuron activity is perturbed before the detection of NMJ abnormalities, and that these circuit abnormalities correlate with impairments in motor behaviors (Mentis et al., 2011). Data are lacking for quantifying the effect of loss of *C. elegans smn-1* on locomotion or NMJ integrity and the effect of modifier genes on these phenotypes.

We addressed these questions using automated image tracking and processing coupled with hydrodynamic models to extract quantitative kinematic (e.g., swimming speed) and dynamic (e.g., propulsive force) properties of *C. elegans* during swimming in a balanced salt solution (M9; Brenner, 1974). Previously, *smn-1(ok355)* mutant animals have been shown to exhibit a progressive decline in a single measure - thrashing rate after the second larva (L2) stage (Briese et al., 2009). With this in mind, we monitored the swimming behavior of each experimental group at two different stages, L2 and “late” (L2 + 3 d), and determined if a reduction of *daf-2* signaling rescues locomotion deficit in *smn-1(ok355)* mutant animals. Choosing the best comparison groups for the late stage is not straightforward; late *smn-1(ok355)* mutant animals cannot be compared with 5 days-old N2 animals because they have arrested at an earlier larval stage. We selected control groups based on the most biomechanically relevant developmental parameter, the body length. The typical body lengths for the two experimental groups are 0.39 ± 0.01 mm (L2) and 0.63 ± 0.08 mm (late; Table 2).

**Table 2.** Biomechanical profiling of N2, *daf-2(e1370)*, *smn-1(ok355)*, and *smn-1(ok355);daf-2(e1370)* at two developmental stages

	L2 stage^*^				Late stage^#^			
BMP output	N2 (n = 22)	*daf-2(e1370)* (n = 24)	*smn-1(ok355)* (n = 28)	*smn-1(ok355);* *daf-2(e1370)* (n = 25)	N2 (n = 13)	*daf-2(e1370)* (n = 15)	*smn-1(ok355)* (n = 20)	*smn-1(ok355);* *daf-2(e1370)* (n = 25)
Length (mm)	0.39 ± 0.01	0.43 ± 0.03	0.48 ± 0.07	0.45 ± 0.03	0.63 ± 0.08	0.70 ± 0.03	0.70 ± 0.03	0.61 ± 0.07
Swimming speed (mm/s)	0.16 ± 0.06	0.17 ± 0.04	0.19 ± 0.06	0.19 ± 0.05	0.26 ± 0.07	0.28 ± 0.07	0.16 ± 0.04	0.18 ± 0.04
Beating frequency (Hz)	1.90 ± 0.39	2.20 ± 0.42	2.50 ± 0.52	2.60 ± 0.39	2.30 ± 0.25	2.30 ± 0.46	1.70 ± 0.36	2.30 ± 0.39
Bending force (nN)	0.50 ± 0.21	0.36 ± 0.09	0.50 ± 0.18	0.42 ± 0.19	0.69 ± 0.27	0.66 ± 0.19	0.50 ± 0.18	0.52 ± 0.11
Mechanical power (pW)	0.50 ± 0.24	0.57 ± 0.16	0.88 ± 0.40	0.60 ± 0.30	1.80 ± 0.45	1.70 ± 0.57	0.97 ± 0.39	1.21 ± 0.37

* Each respective worm is at L2 stage.

# Late stage: N2 and *daf-2(e1370)* animals are at L4 stage; *smn-1(ok355)* and *smn-1(ok355);daf-2(e1370)* animals are at the day 5 after egg drop (L2 + 3 d).

**Figure 3.**
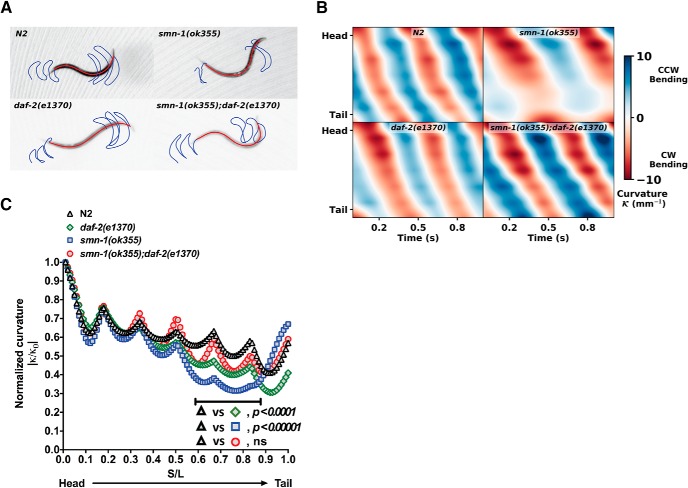
Reduced *daf-2* signaling rescues the body curvature of *smn-1(ok355)* animals during swimming. ***A***, A trace of the head and tail trajectories for typical individuals from each genotype (e.g., N2, *smn-1(ok355)*, *daf-2(e1370)* and *smn-1(ok355);daf-2(e1370)*). There is no qualitative difference between N2 versus the *daf-2(e1370)* mutant animals while the *smn-1(ok355)* mutant animals swim more slowly and beat less frequently than N2 worms. The *smn-1(ok355);daf-2(e1370)* mutant animals closely resemble N2 worms. ***B***, Curvature kymographs of representative individuals of each genotype reveals a common pattern shared by N2, *daf-2(e1370)*, and *smn-1(ok355);daf-2(e1370)* mutant animals. In contrast, *smn-1(ok355)* mutant animals exhibits relatively little curvature (white color) compared to other genotypes at a region from approximately the middle of the body to just before the tail, implicating a regional loss of neuromuscular function. CW, clockwise; CCW, counterclockwise. ***C***, Quantification of regional body curvature in *smn-1* mutant animals. We quantified the body curvature of animals with four genotypes (N2, *smn-1(ok355)*, *daf-2(e1370)*, and *smn-1(ok355);daf-2(e1370)*) and find that the curvature of each group of worms appears to have local maxima when averaged over time (for details, see Extended Data Fig. 3-1). For the N2 and *daf-2(e1370)* mutant animals, the mean normalized curvature decreases from head to tail. The *daf-2(e1370)* group shows a significant low curvature at the tail region compared to the N2 group. The *smn-1(ok355)* group has a distinct curvature pattern. Its curvature decreases rapidly after the worm’s mid-point and then increases sharply near the tail. This pattern corresponds to the region of low curvature observed in Figure 3*B*. In the *daf-2* mutant background (*smn-1(ok355);daf-2(e1370)*), body curvature during swimming is normalized and appears similar to N2 and *daf-2(e1370*) mutant animals. This is quantitative evidence that the *daf-2* mutant background rescues the neuromuscular defect seen in the midsection and tail of the *smn-1(ok355)* mutant animals. ns, no significance.

Simple observation of the swimming gait of individuals from each genotype over one second demonstrates that *smn-1(ok355)* mutant animals move slowly and beat less frequently than N2 and *daf-2(e1370)* mutant animals (Fig. 3*A*). In the *daf-2* mutant background, *smn-1(ok355)* mutant animals demonstrate a qualitative improvement in swimming gait, leading to a pattern that more closely resemble N2 animals. Next, we present kymographs of the worm’s body curvature over one second of forward swimming (Fig. 3*B*; for more detail, see Materials and Methods). The stripes show body curvature progressing down the length of the animal from head to tail; the slope of these stripes represent the wave speed, or the rate at which waves of curvature progress along the body of the worm. The rate at which the pattern repeats itself represents the frequency f of the worms beating. When examining the curvature kymographs of each genotype, we see that *smn-1(ok355)* mutant animals have a region along their body near the tail that exhibits no curvature. This region stretches from approximately the middle of the worm to just before the tail ($0.5<\frac{s}{L}<0.8)$. This suggests a LOF of neuromuscular function in that region, since repeated contraction and relaxation would bend the worm locally, therefore producing curvature. The “failure of curvature” phenotype is reversed in the *daf-2* mutant background (i.e., animals with the *smn-1(ok355);daf-2(e1370)* genotype).

10.1523/ENEURO.0289-18.2018.f3-1 Extended Data Figure 3-1 **Statistical analysis of normalized body curvature in N2, *smn-1(ok355)*, *daf-2(e1370)*, and *smn-1(ok355);daf-2(e1370)* mutant animals. **Statistical analysis for normalized body curvature data shown in Figure 3*C*. Multiple *t* tests followed by Holm–Sidak method correction for significant; * reach to statistical significance. Download Figure 3-1, TIF file.

To interrogate this failure of curvature issue more closely, we recall that the body curvature of the nematode is dependent on its local orientation and its parameterized body shape. By quantifying the body curvature of all the nematodes for each genotype, we systematically probe this aspect of worm locomotion with high sensitivity. The curvature of a worm appears to have nodes when we take an average in time over each population (Fig. 3*C*; Extended Data Fig. 3-1). For the N2 and *daf-2(e1370)* mutant animals, we observed the mean normalized curvature decreases from head to tail. The *daf-2* group shows a significant lower curvature at its tail region compared to the N2 group. The curvature pattern in the *smn-1(ok355)* mutant animals are very distinct from N2 and *daf-2*. It decreases rapidly after the worm’s mid-point and then sharply increase right near the tail (Fig. 3*C*; adjusted *p* < 0.00001 by multiple *t* tests and Holm–Sidak method correction). This pattern corresponds to the region of low curvature observed in Figure 3*B* kymographs. Moreover, we observed that *smn-1(ok355)*;*daf-2(e1370)* mutant animals displayed a normalized mean curvature between those of N2 and *daf-2(e1370)* mutant animals. It is clear that the defects in body curvature in the midsection and tail incurred by loss of *smn-1* are rescued in the *daf-2* mutant background.

**Figure 4.**
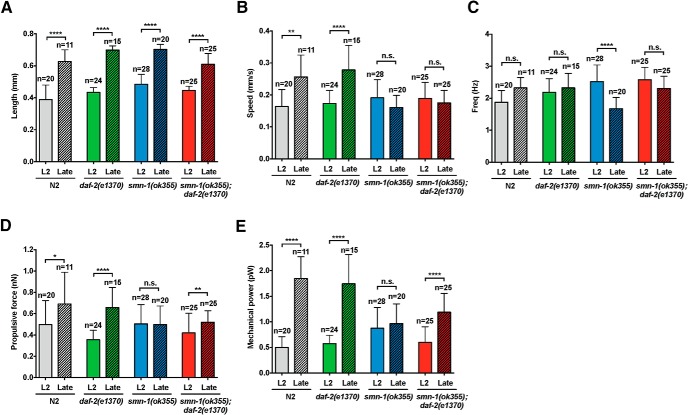
Statistical analysis of biomechanical properties (BMP). Bar graphs represent BMP algorithm calculated (***A***) length (mm), (***B***) speed (mm/s), (***C***) frequency (Hz), (***D***) propulsive force (nN), and (***E***) mechanical power (pW) for the indicated genotypes at L2 or late time points. Data shown are mean ± SD, ≥10 animals per genotype were recorded. Nonparametric Kruskal–Wallis test followed by Dunn’s multiple comparison test for significant; * *p* < 0.05, ** *p* < 0.01, ***p < 0.005, ****p < 0.001, ns, no significance. ***A***, Genotypes are well matched for length at the two time points. ***B***, Age-dependent increase in speed was shown in both N2 and *daf-2(e1370)* mutant animals but not in *smn-1(ok355)* or *smn-1(ok355);daf-2(e1370)* mutant animals. ***C***, Beat frequency diminishes over time in the *smn-1(ok355)* mutant animals and this is suppressed in the *smn-1(ok355);daf-2(e1370)* mutant animals. ***D***, Age-dependent increase in propulsive force is seen in N2 and *daf-2(e1370)* mutant animals; this is not seen in the *smn-1(ok355)* mutant animals, but is rescued in the *smn-1(ok355);daf-2(e1370)* mutant animals. ***E***, Age-dependent increase in mechanical power force is seen in N2 and *daf-2(e1370)* mutant animals; this is not seen in the *smn-1(ok355)* animals but is rescued in the *smn-1(ok355);daf-2(e1370)* mutant animals.

Next, we undertook a quantitative analysis of worm swimming using in-house biomechanical profiling algorithms first published in *Genetics* (Krajacic et al., 2012), and examined 13.5 h of videos at 30 frames per second (approximately yielding 570 gigabytes of data). Using prespecified criteria to exclude substandard data (as described in Materials and Methods), we ultimately processed 0.76 h of videos (corresponding to 31.9 gigabytes of data). Figure 4*A* shows that the body length of each group is significantly increased from L2 to late stage. There is no significant difference in body length across all groups at each stage (L2 or late stage; Fig. 4*A*; Table 2). The body length is the most biomechanically relevant developmental parameter and serves as the control here. Then, we begin evaluated various biomechanical properties by swimming assay. First, we measured each experimental group’s swimming speed U and beating frequency f. At the L2 stage, the swimming speed of N2 animals is ∼0.16 mm/s (Fig. 4*B*), and this is no different from *daf-2(e1370*), *smn-1(ok355*), and *smn-1(ok355);daf-2(e1370*) mutant animals (*p* > 0.05, Kruskal–Wallis test). With developmental progression (i.e., the late stage), wild-type N2 and *daf-2(e1370*) animals significantly increase their swimming speed by 63% and 65%, respectively. In contrast, at the late stage s*mn-1(ok355)* and *smn-1(ok355);daf-2(e1370)* mutant animals trend toward a decrease in swimming speed, there was no statistically significant difference between swimming speed at the early and late time points. This suggests that the loss of s*mn-1* impairs the normal, age-dependent increase in locomotion rate.

Next, we investigated beating frequency in these animals. Although N2 animals increases modestly over time (1.9 Hz at the L2, and 2.3 Hz at the late stage, *p* > 0.05, Kruskal–Wallis test), both N2 and *daf-2(e1370)* animals did not show significant increase in frequency (Fig. 4*C*). The beating frequency of *smn-1*(*ok355*) mutant animals at the L2 stage is significantly higher than N2-L2 animals (2.5 vs 1.9 Hz, *p* < 0.05, Kruskal–Wallis test) and decreases by 32% at the late stage (e.g., 2.3–1.7 Hz, *p* < 0.01, Kruskal–Wallis test). We also found that the beating frequency of *smn-1(ok355);daf-2(e1370)* mutant animals at the L2 stage is similar to the age-matched *smn-1(ok355)* (2.6 vs 2.5 Hz). Interestingly, unlike the *smn-1(ok355*) group, *smn-1(ok355);daf-2(e1370)* mutant animals are able to maintain beating frequency at the late stage in a fashion similar to wild-type N2. A pathologic decrement in neuromuscular activity over time in the *smn-1(ok355)* mutant animals could account for the observed decrease in beat frequency. Such pathologic decrement does not occur in the *smn-1(ok355);daf-2(e1370)* mutant animals.

The analysis of these simple swimming kinematics reveals a pathologic beating frequency in the *smn-1(ok355)* mutant animals, this does not occur in the *smn-1(ok355);daf-2(e1370)* mutant animals (e.g., the swimming speed over time is unmodified). While swimming frequency and speed are important factors in describing locomotion, there are of other determinants that contribute to propulsive force and mechanical power. We used the hydrodynamic model resistive force theory (Gray and Hancock, 1955) to estimate the propulsive force and mechanical power of *C. elegans*, measurements that more directly probe muscle output and the energy expended during locomotion.

We expect that as a worm develops, the propulsive force of the organism will increase. Indeed, we found that both N2 and *daf-2(e1370)* animals exhibited an increase in propulsive force of 39% and 83%, respectively, when they progress from L2 to late stage (*p* < 0.01, Kruskal–Wallis test; Fig. 4*D*). In contrast, for *smn-1(ok355*) mutant animals, the propulsive force remains the same during this developmental period, suggesting that motor output is impaired. In contrast, *smn-1(ok355*);*daf-2(e1370*) mutant animals show an increase in propulsive force by 24% (*p* < 0.01, Kruskal–Wallis test) over this period. Thus, the normal increase in motor output as worms mature fails to occur in *smn-1* mutant animals. Reduced *daf-2* signaling can rescue this defect.

Next, we investigate the mechanical power expended by each group during swimming. We observed that N2 and *daf-2(e1370)* animals significantly increase their mechanical power (250% and 200%, respectively) as they develop (Fig. 4*E*). In contrast, *smn-1(ok355)* mutant animals produced the same mechanical power at the L2 and the late stage; in addition, the mechanical power of the *smn-1(ok355)* group is significantly lower than N2, late stage (0.9 vs 1.7 pW, *p* < 0.001, Kruskal–Wallis test). Reduction of *daf-2* signaling (*smn-1(ok355*)*;daf-2(e1370)*) significantly improves mechanical power when compared to *smn-1(ok355*) mutant animals. Mechanical power for locomotion of *smn-1(ok355*)*;daf-2(e1370)* mutant animals is statistically indistinguishable from that of the late stage wild-type group (*p* > 0.05, Kruskal–Wallis test; Fig. 4*E*).

In summary, the kinematic and dynamic biomechanical properties (i.e., frequency, propulsive force, and mechanical power) show that a reduction of *daf-2* signaling improves several aspects of locomotor capability in *smn-1(ok355)* mutant animals. While these modifications do not directly increase the swimming speed of the organism, they improved the beating patterns, muscle output, and the energy expended during locomotion.

### Reduced *daf-2* signaling preserves the postsynaptic morphology

**Figure 5.**
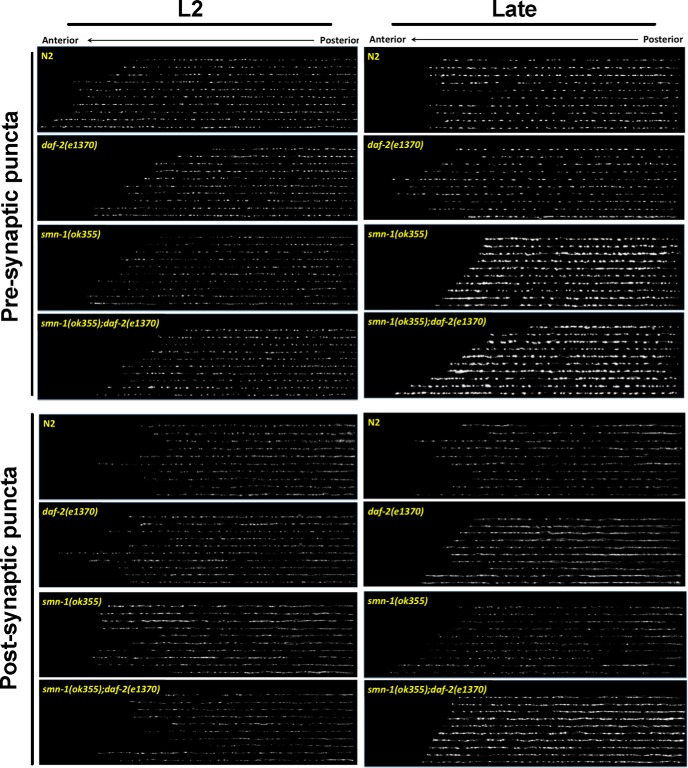
Morphologic examination of presynapse and postsynapse in *smn-1* mutant animals. ***A***, top, Confocal images of GFP expression in the presynaptic terminal of GABAergic motor neurons [*synaptobrevin::GFP* driven by *unc-25* (GAD) promoter] in L2 and Late (L2 + 3 d) animals (four genotypes: N2, *smn-1(ok355)*, *daf-2(e1370)* and *smn-1(ok355);daf-2(e1370)*). Bottom, Confocal images of GFP expression in postsynaptic GABAergic neuromuscular synapses [*UNC-49::GFP* driven by *unc-49* (GABA receptor) promoter] in L2 and Late (L2 + 3 d) animals. All images were obtained from same region of dorsal nerve cord of the *C. elegans* strains and each line of puncta images are derived from a single animal as a spatial raster plot.

**Figure 6.**
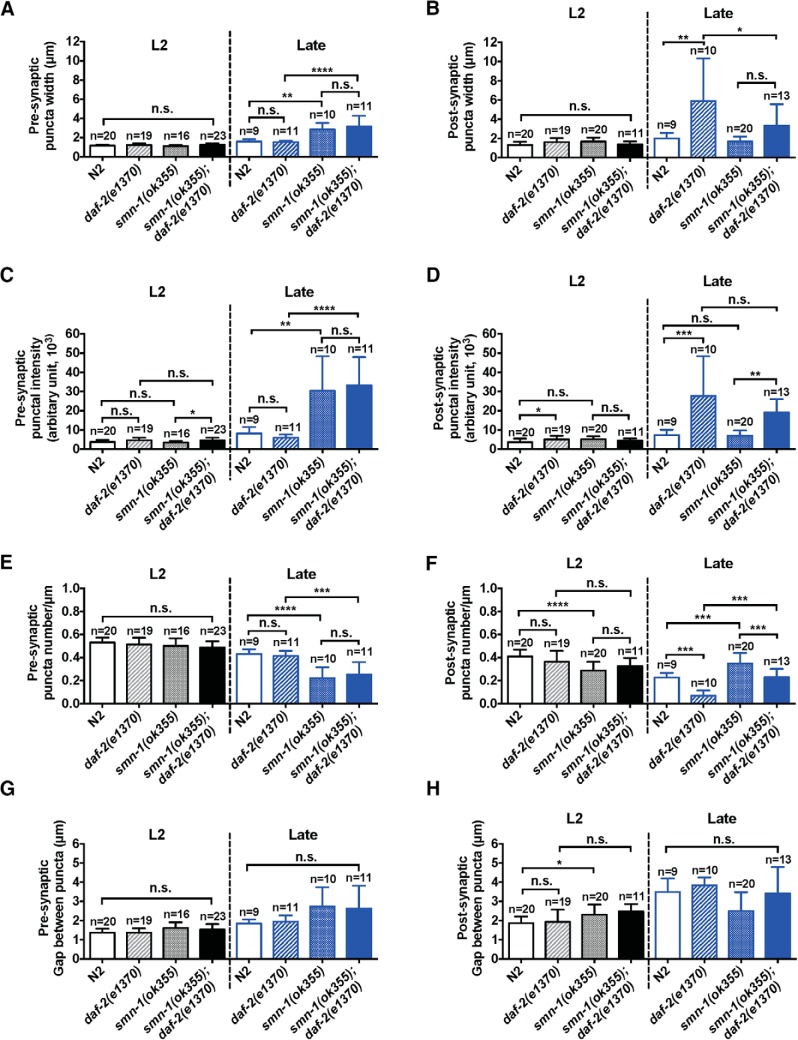
Quantification of puncta analysis reveals GABAergic synaptic defects in *smn-1* mutant animals are partially rescued by reduction of *daf-2* activity. ***A***, ***B***, Quantification of puncta width in presynapse and postsynapse at L2 or late stage in different genotypes of worm. ***C***, ***D***, Quantification of puncta intensity in presynapse and postsynapse at L2 or late stage in different genotypes of worm. ***E***, ***F***, Quantification of puncta number in presynapse and postsynapse at L2 or late stage in different genotypes of worm. ***G***, ***H***, Quantification of gap between puncta in presynapse and postsynapse at L2 or late stage in different genotypes of worm. *p < 0.05; **p < 0.01; ***p < 0.005; ****p < 0.001; ns, no significance.

To assess the contribution of abnormalities in the NMJ structure to the locomotor defects defined above, we examined the morphology of inhibitory synapses by using a presynaptic (Punc-25/GAD-synaptobrevin::GFP fusion protein) and postsynaptic [Punc-49/GABA receptor subunit (UNC-49B)::GFP fusion protein] marker (Fig. 5). To quantify synaptic morphology defects, we analyzed the puncta width, density (number of puncta per micrometer), fluorescence intensity, and the gap between puncta along the dorsal cord using ImageJ and PunctaAnalyzer program (Fig. 6; Hung et al., 2007). Four experimental groups were compared: (1) presynaptic or postsynaptic reporter in the N2 background, (2) presynaptic or postsynaptic reporter in the *daf-2(e1370)* background, (3) presynaptic or postsynaptic reporter in the *smn-1* mutant background, and (4) presynaptic or postsynaptic reporter in the *smn-1(ok355);daf-2(e1370)* background.

At the L2 stage, a qualitative comparison of presynaptic marker revealed essentially no differences in puncta localization and distribution among the four experimental groups (Fig. 5, top left). The quantitative analysis confirms this with the only exception that puncta intensity in L2 stage of *smn-1(ok355);daf-2(e1370*) mutant animals was 33% stronger than *smn-1* mutant animals (*p* < 0.05; Fig. 6*A*,*C*,*E*,*G*). A qualitative comparison of postsynaptic marker at L2 stage reveals a few small group differences in puncta localization and distribution among the four groups (Fig. 5, bottom left). Quantitatively, the most pronounced difference was that *smn-1(ok355)* mutant animals had 30% fewer puncta (Fig. 6*F*) and concomitantly larger gaps between these puncta when compared with N2 group (Fig. 6*H*). Reduced *daf-2* signaling did not rescue this phenotype. Overall, at the L2 stage, all groups exhibit similar presynaptic and postsynaptic puncta patterns although a few, relatively subtle, group differences are evident.

At the later developmental point, a qualitative comparison of presynaptic puncta revealed substantial differences in puncta distribution between the four groups (Fig. 5, top right). Quantitatively the puncta of *smn-1(ok355)* and *smn-1(ok355)*;*daf-2(e1370*) mutant animals were significantly (∼100%) wider than controls (N2 or *daf-2(e1370)* mutant animals, *p <* 0.01 for N2 vs *smn-1(ok355)* and *p* < 0.001 for *daf-2(e1370*) vs *smn-1(ok355);daf-2(e1370*), one-way ANOVA followed by *post hoc* Tukey HSD for multiple comparisons; Fig. 6*A*), but there were fewer of them (48% less dense in *smn-1(ok355)* than in N2, *p* < 0.001; 39% less dense in *smn-1(ok355);daf-2(e1370)* than in *daf-2(e1370*), *p* < 0.005, one-way ANOVA with Tukey test; Fig. 6*E*). In addition, puncta intensity in *smn-1(ok355)* and *smn-1(ok355);daf-2(e1370)* mutant animals are ∼300% greater than in N2 or *daf-2(e1370)* (*p* < 0.01 for N2 vs *smn-1(ok355)* and *p* < 0.001 for *daf-2(e1370)* vs *smn-1(ok355);daf-2(e1370*), one-way ANOVA with Tukey test; Fig. 6*C*). No differences in puncta intensity were seen when N2 versus *daf-2(e1370)* animals were compared or when *smn-1(ok355)* versus *smn-1(ok355);daf-2(e1370)* mutant animals were compared. Thus, over development, presynaptic GABAergic terminals of *smn-1(ok355)* mutant animals appear to be larger and have undergone structural reorganization. A reduction in *daf-2* signaling appears to have no impact on this phenotype.

A qualitative comparison of postsynaptic puncta also revealed substantial differences in puncta localization and distribution between the four groups (Fig. 5, bottom right). Quantitatively, we found that *daf-2(e1370)* animals have ∼2-fold wider and 2.7-fold brighter puncta on body wall muscles compared to N2 animals (*p* < 0.005 for both width and puncta intensity, one-way ANOVA with Tukey test), and this is associated with a reduction in puncta number (*p <* 0.005, one-way ANOVA Tukey test; Fig. 6*F*). We also found that reduction of *daf-2* signaling enhances GABAergic puncta intensity in *smn-1(ok355)* mutant animals (Fig. 6*D*). While puncta width was the same in *smn-1(ok355);daf-2(e1370)* and *smn-1(ok355)* mutant animals (Fig. 6*B*), s*mn-1(ok355);daf-2(e1370)* mutant animals have ∼300% brighter puncta than in the *smn-1(ok355)* mutant animals (*p* < 0.01, one-way ANOVA with Tukey test; Fig. 6*D*). Reduced *daf-2* signaling in *smn-1(ok355)* mutant animal leads to a large increase in GABA receptor content at/or near synapses (Fig. 6*B*,*D*).

Together, these observations reveal that young *smn-1(ok355)* mutant animals appear to have a normal GABAergic NMJs. Over time there are two major changes to these synapses: (1) presynaptic elements decrease in number but are restructured, and (2) the number of postsynaptic elements increase without an effect on width or GABAergic receptor content. The main effect of *daf-2* reduction is to decrease postsynaptic element number to the N2 level and to increase the synaptic content of GABA receptors. It is difficult to predict the functional consequence of these diametrically opposing effects of the *daf-2* mutant animals on postsynaptic GABA receptors.

### Postsynaptic defects in GABAergic NMJs significantly contribute to *smn-1(ok355)*’s hypersensitivity to pyridostigmine bromine

To investigate the functional consequence of morphologic abnormality of GABAergic NMJs observed above, we used pharmacological tools: pyridostigmine bromide (Sleigh et al., 2011), an acetylcholinesterase inhibitor, and levamisole, a cholinergic agonist. The time course of hypercontractive paralysis of animals bathed in these agents can report the balance between muscle excitatory cholinergic and inhibitory GABAergic transmission (Mahoney et al., 2006; Dabbish and Raizen, 2011). We examined animal sensitivity to levamisole or pyridostigmine bromide at two time points: L2 stage and late stage (Fig. 6). Where applicable, we classified animals as resistant to inhibitors of cholinesterase (Ric) or hypersensitive to inhibitors of cholinesterase (Hic).

**Figure 7.**
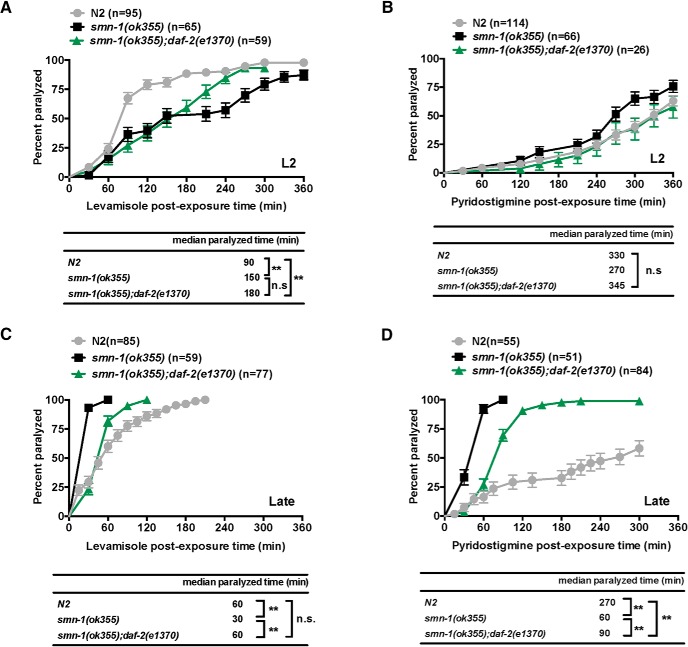
Loss of *smn-1* influences sensitive to pyridostigmine and levamisole. ***A***, ***B***, L2 animals of genotype N2*, smn-1(ok355)* or *smn-1;daf-2(e1370)* were subjected to the levamisole or pyridostigmine sensitivity test. At this age, *smn-1(ok355)* and *smn-1;daf-2(e1370)* mutant animals resisted levamisole greater than N2 animals. ***C***, ***D***, Late stage (L2 + 3 d) animals of genotype N2, *smn-1(ok355)*, or *smn-1;daf-2(e1370)* were also subjected to levamisole or pyridostigmine sensitivity test. At the late stage, *smn-1(ok355)* but not *smn-1(ok355);daf-2(e1370)* mutant animals are hypersensitive to levamisole. In addition, *smn-1(ok355)* and *smn-1(ok355);daf-2(e1370)* mutant animals show hypersensitivity to the pyridostigmine bromide although *daf-2(e1370)* mutant animals suppresses the degree of hypersensitivity conferred by *smn-1(ok355)* mutant animals. Data points represent the mean ± SEM percentage of animals paralyzed at 30-min intervals over a period of 6 h; **p < 0.001; ns, no significance, Log-rank test.

At the L2 stage, both *smn-1(ok355)* and *smn-1(ok355);daf-2(e1370)* mutant animals exhibited a levamisole-resistance phenotype (Fig. 7*A*). The median paralyzed time of *smn-1(ok355)* and *smn-1(ok355);daf-2(e1370)* mutant animals are ∼66% and 100%, respectively, longer than the time in the wild-type N2 animals (*p* < 0.01, Log-rank test). There is no significant difference between *smn-1(ok355)* and *smn-1(ok355);daf-2(e1370).* Thus, *smn-1(ok355)* mutant animals are resistant to direct cholinergic agonists. When we assayed animal’s sensitivity to pyridostigmine bromide, however, we found no significant differences between wild-type N2, *smn-1(ok355)*, and *smn-1(ok355);daf-2(e1370)* at L2 stage (Fig. 7*B*). These observations suggest that animals with the *smn-1(ok355)* genotype have a shift in the balance between excitatory and inhibitory neurotransmission toward decreased excitability. Either a reduction in cholinergic neurotransmission or an enhancement in GABAergic neurotransmission (or a combination of both) would be compatible with these observations

Interestingly, at the late stage, we observed a sensitivity switch to levamisole from resistance to hypersensitivity in *smn-1(ok355)* mutant animals (Fig. 7*C*). The median time to paralysis of *smn-1(ok355)* is 50% shorter than the time in the N2 (*p* < 0.01, Log-rank test). Loss of daf-2 signaling rescues this phenotype; the levamisole sensitivity of *smn-1(ok355);daf-2(e1370)* mutant animals is the same as N2 animals. For pyridostigmine test in late stage of animals, both *smn-1(ok355)* and *smn-1(ok355);daf-2(e1370)* mutant animals show hypersensitivity phenotype (Fig. 7*D*). *smn-1(ok355)* and *smn-1(ok355);daf-2(e1370)* mutant animals are 3.5-fold and 2-fold shorter time course to reach the median paralyzed time when compared to wild-type N2 (*p* < 0.01, Log-rank test). The *smn-1(ok355);daf-2(e1370)* mutant animal are slightly less hypersensitive to pyridostigmine than the *smn-1(ok355)* mutant animals (Fig. 7*D*). These observations suggest that *smn-1(ok355)* mutant animals have now shifted the balance between excitatory and inhibitory neurotransmission toward increased excitability (e.g., they are Hic). Either an enhancement in cholinergic neurotransmission or a reduction in GABAergic neurotransmission (or a combination of both) would be compatible with these observations. That the HIC phenotype is suppressed in the *daf-2(e1370)* background suggests that equipoise in the excitation/inhibition balance has been normalized.

To the extent that changes in GABAergic transmission are contributing to these observations, we interpret our morphologic observations to indicate that: (1) reduced GABAergic transmission in *smn-1(ok355)* mutant animals is predominantly due to reduced number of presynaptic elements, and (2) benefit of reduced *daf-2* in the *smn-1(ok355)* mutant animals is due to increased density of GABA receptors at synapses.

### Enhancing GABAergic transmission improves locomotion of *smn-1(ok355)* mutant animals

If the locomotor benefit conferred on *smn-1(ok355)* mutant animals by loss of *daf-2* is mainly due to increased GABAergic transmission, a functional enhancement of the GABAergic system in the *smn-1(ok355)* mutant animals should phenocopy loss of *daf-2* function. To test this idea, we identified two strains of worms that are likely to exhibit enhanced GABAergic neurotransmission without affecting the baseline swimming behaviors: RM2710 (*snf-11(ok156)*) and FY297 (*oxls22(UNC-49::GFP)*). The *snf-11(ok156)* mutant animals lack the sodium-dependent GABA transporter (which clears synaptic GABA). These animals are aldicarb- resistant supporting the view that they have enhancement of GABAergic transmission (Mullen et al., 2006). The overexpression of multiple subunits of the GABA receptors in *oxls22(UNC-49::GFP)* animals probably have enhanced GABAergic transmission, although to our knowledge, this has never been formally tested.

**Figure 8.**
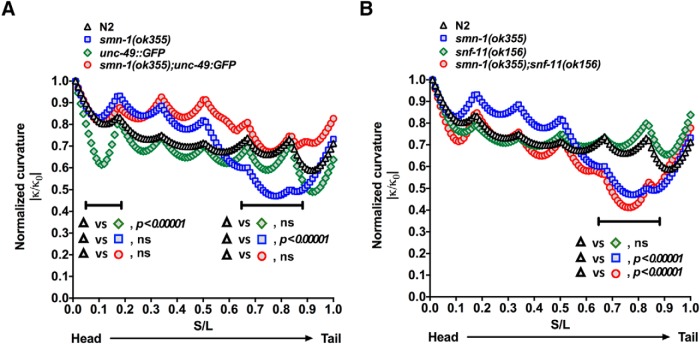
*smn-1(ok355)* mutant animals have an abnormal body curvature during swimming that is rectified by reduced *UNC-49::GFP* overexpression but unaffected by *snf-11(ok156)*. ***A***, The body curvature of animals with four genotypes (N2, *smn-1(ok355), oxls22(UNC-49::GFP)*, *smn-1(ok355)*;*oxls22(UNC-49::GFP)*) were quantified (for details, see Extended Data Fig. 8-1). For the N2 and *oxls22(UNC-49::GFP)* mutant animals, the mean normalized curvature decreases from 1 (by definition) to ∼0.5 and 0.4 (head to tail orientation), respectively. The *smn-1(ok355)* group has a fundamentally different shape, decreasing rapidly after the worm’s mid-point to 0.4, and then sharply increasing near the tail; this decrease in curvature corresponds to the region of low curvature observed in Figure 3*B*,*C*. In the *oxls22(UNC-49::GFP)* mutant background (*smn-1(ok355);oxls22(UNC-49::GFP)* body curvature during swimming is normalized and appears similar to N2 and *oxls22(UNC-49::GFP)* mutant animals. This result is very similar to observations noted above (Fig. 3*C*) on the effect of the *daf-2* mutant background on the neuromuscular defect seen in the midsection and tail of the *smn-1(ok355)* mutant animals. ***B***, The body curvature of animals with four genotypes (N2, *smn-1(ok355)*, *snf-11(ok156)*, *smn-1(ok355)*;*snf-11(ok156)*) were quantified (for details, see Extended Data Fig. 8-2). For the N2 and *snf-11(ok156)* mutant animals, the mean normalized curvature decreases from 1 (by definition) to ∼0.5 and 0.4 (head to tail orientation), respectively. The *smn-1(ok355)* group has a fundamentally different shape, decreasing rapidly after the worm’s mid-point to 0.4, and then sharply increasing near the tail; this decrease in curvature corresponds to the region of low curvature observed in Figures 3*B*,*C*-7*A*. In the *snf-11(ok156)* mutant background, the body curvature of *smn-1(ok355);snf-11(ok156*) mutant animals during swimming remains abnormal and is indistinguishable from the swimming *of smn-1(ok355)* mutant animals. ns, no significance.

We began by quantifying body curvature of following strains: N2, *smn-1(ok355)*, *oxls22(UNC-49::GFP)*, and *smn-1(ok355);oxls22(UNC-49::GFP)*. The mean normalized curvature (from head to tail) in N2 and *oxls22(UNC-49::GFP)* are similar, except the head region (0.07–0.13% of body length from the head) of *oxls22(UNC-49::GFP)* group, which shows a significantly lower curvature than N2 (Fig. 8*A*; Extended Data Fig. 8-1; by multiple *t* tests). Consistent with the data observed previously (Fig. 3*A*,*C*), the *smn-1(ok355)* mutant animals display a significantly small curvature at the tail region (0.68–0.87% of body length from the head) compared with N2 (Fig. 8*A*; adjusted *p* < 0.00001 by multiple *t* tests and Holm–Sidak method correction). Interestingly, the normalized mean curvature of *smn-1(ok355)*;*oxls22(UNC-49::GFP)* animals shows no significant difference to those of N2 and *UNC-49::GFP* animals (Fig. 8*A*).

10.1523/ENEURO.0289-18.2018.f8-1 Extended Data Figure 8-1 **Statistical analysis of normalized body curvature in *smn-1(ok355);unc-49::GFP* mutant animals.** Statistical analysis for normalized body curvature data of *smn-1(ok355);unc-49::GFP* mutant animals shown in Figure 8*A*. Multiple *t* tests followed by Holm–Sidak method correction for significant; * reach to statistical significance. Download Figure 8-1, TIF file.

10.1523/ENEURO.0289-18.2018.f8-2 Extended Data Figure 8-2 **Statistical analysis of normalized body curvature in *smn-1(ok355);snf-11(ok156)* mutant animals.** Statistical analysis for normalized body curvature data of *smn-1(ok355);snf-11(ok156)* mutant animals shown in Figure 8*B*. Multiple *t* tests followed by Holm–Sidak method correction for significant; * reach to statistical significance. Download Figure 8-2, TIF file.

Next, we analyzed the curvature in the second set of animals. The four experimental groups were: N2, *smn-1(ok355)*, *snf-11(ok156)*, and *smn-1(ok355);snf-11(ok156).* Both N2 and *snf-11(ok156)* animals show very similar curvature pattern (Fig. 8*B*). In contrast, the *smn-1(ok355)* mutant animals display a distinct curvature shape. The normalized mean curvature in *smn-1(ok355)* animals decreases rapidly after the worm’s mid-point and then sharply increases right near the tail. The *smn-1(ok355)*;*snf-11(ok156)* mutant animals display a curvature pattern similar to *smn-1(ok355)* mutant animals but distinct from the N2 and *snf-11(ok156)* mutant animals, especially from the mid-point to tail region (Fig. 8*B*; Extended Data Fig. 8-2; adjusted *p* < 0.00001 by multiple *t* tests and Holm–Sidak method correction). This result indicates that loss of the GABA uptake transporter (i.e., *snf-11(ok156)*) does not improve the curvature phenotype of the *smn-1(ok355)* mutant animals (Fig. 8*B*). These two means of enhancing GABAergic neurotransmission have non-identical effects on network behavior, a point that will be addressed further in Discussion.

**Figure 9.**
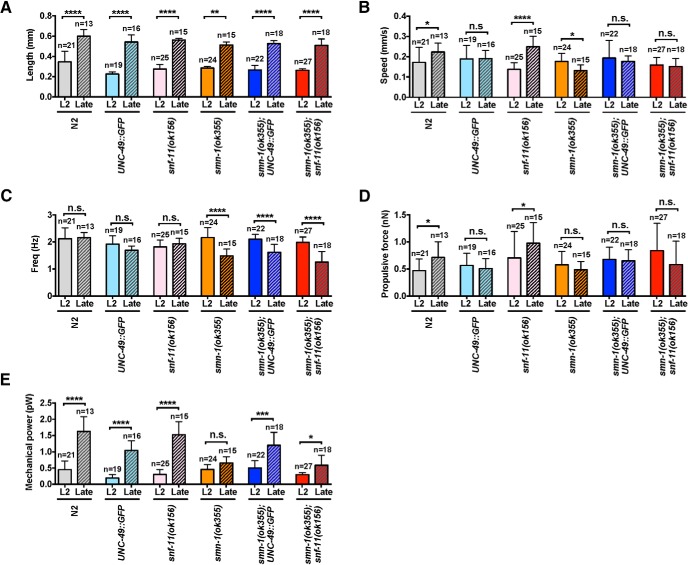
Statistical analysis of biomechanical properties (BMP). Bar graphs represent BMP algorithm calculated (***A***) length (mm), (***B***) speed (mm/s), (***C***) frequency (Hz), (***D***) propulsive force (nN), and (***E***) mechanical power (pW) for the indicated genotypes from L2 or late stage. Data are shown as mean ± SD; ≥10 animals per genotype were recorded. Nonparametric Kruskal–Wallis test followed by Dunn’s multiple comparison test for significant; * *p* < 0.05, ** *p* < 0.01, ***p < 0.005, ****p < 0.001, ns, no significance. ***A***, Genotypes are well matched for length at the two time points. ***B***, N2 and *snf-11(ok156)* animals show age-dependent increase while the *smn-1(ok355)* mutant animals show a decrease in speed. This decrease in speed is suppressed in the *smn-1(ok355)*;*oxls22(UNC-49::GFP)* and *smn-1(ok355);snf-11(ok156)* mutant animals. ***C***, Beat frequency remains constant over time in the N2, *oxls22(UNC-49::GFP)*, and *snf-11(ok156)* mutant animals. It declines in the *smn-1*(*ok355*), *smn-1(ok355)*;*oxls22(UNC-49::GFP)*, and *smn-1(ok355);snf-11(ok156)* groups. ***D***, Age-dependent increase in propulsive force is seen only in the N2 and *snf-11(ok156)* mutant animals. ***E***, Age-dependent increase in mechanical power force is seen in N2, *oxls22(UNC-49::GFP)* and *snf-11(ok156)* but not in the *smn-1(ok355)* mutant animals. Age-dependent increase in mechanical power force is restored in the *smn-1(ok355);oxls22(UNC-49::GFP)* and *smn-1(ok355);snf-11(ok156)* mutant animals.

Furthermore, we undertook a quantitative analysis of their kinematic and biomechanical properties (Fig. 9). Between L2 and late stage, all animals grew to a statistically significant increase in length (Fig. 9*A*). While N2 and s*nf-11(ok156)* mutant animals display a progressive increase in swimming speed between L2 and late stage, the *smn-1(ok355)* mutant animals display a significant decrease in swimming speed. This decrement in swimming speed was prevented in the *smn-1(ok355)*;*oxls22(UNC-49::GFP)* and *smn-1(ok355)*;*snf-11(ok156)* mutant animals (Fig. 9*B*). There is no change in beating frequency between L2 and late stage in animals with genotype N2, *oxls22(UNC-49::GFP)* or *snf-11(ok156)*, while beating frequency declines over this period in *smn-1(ok355)*, *smn-1(ok355)*;*oxls22(UNC-49::GFP)*, and *smn-1(ok355)*;*snf-11(ok156)* mutant animals (Fig. 9*C*). In sum, these studies show that *smn-1(ok355)* animals have an age-dependent decline in swimming speed and beat frequency. Enhancing GABAergic neurotransmission in these animals selectively abrogates the swimming speed defect.

Next, we studied the propulsive force and mechanical power of these animals. With regard to propulsive force, only N2 and *snf-11(ok156)* mutant animals show an increase over the time (L2 vs late stage; Fig. 9*D*). With regard to mechanical power, N2, *oxls22(UNC-49::GFP)* and s*nf-11(ok156)* animals display a progressively significant increase between L2 and late stage, while the *smn-1(ok355)* mutant animals do not display this age-dependent change. In contrast, the *smn-1(ok355)*;*oxls22(UNC-49::GFP)* and *smn-1(ok355)*;*snf-11(ok156)* mutant animals do display a significant age-dependent increase in mechanical power (Fig. 9*E*). In sum, enhanced GABAergic neurotransmission has a selective effect on the dynamic biomechanical properties of the *smn-1(ok355)* mutant animals, it improves their mechanical power but not the propulsive force.

## Discussion

Here, we show that reducing *daf-2*, insulin/IGF signaling (IIS) prolongs life and ameliorates aspects of motor dysfunction in *C. elegans* models of SMA. The effects of reduced *daf-2* signal depend, at least in part, on the *daf-16*, a Forkhead box transcription factor. The *daf-2/daf-16* signaling pathway is conserved in metazoans, and is implicated in the control of human longevity and health-span (Kojima et al., 2004; Suh et al., 2008). Some studies suggest that reduced insulin/IGF signal transduction contributes to the pathophysiology SMA (Hua et al., 2011), and provision of IGF1 is beneficial (Bosch-Marce et al., 2011; Shababi et al., 2011), while other work suggests that reducing levels of the IGF receptor is beneficial (Biondi et al., 2015). Precise control of the level and spatial-temporal regulation of IIS are likely to be critical determinants of efficacy. Here, our results demonstrated that the *daf-2/daf-16* signaling pathway now joins this relatively small number of SMA disease phenotype modifiers, such as plastin 3 (Oprea et al., 2008), RhoA/ROCK (Coque et al., 2014), and four genes in *C. elegans* (*ncbp-2*, *T02G5.3*, *grk-2*, and *flp-4*; Dimitriadi et al., 2010).

In *C. elegans*, previous studies show the reduction of the IIS pathway in several neurodegenerative models by down regulation of *daf-2* can suppress the shorten lifespan and ameliorate the motor deficit (Cohen et al., 2006; Florez-McClure et al., 2007; Zhang et al., 2011; Li et al., 2013). Transgenic worms expressing human Aß_1-42_ in the body wall muscles using the *unc-54* promoter show aggregated-mediate toxicity and age-dependent motor deficit. These disease phenotypes were ameliorated by reducing the activity of the IIS pathway via *daf-2* RNAi feeding (Cohen et al., 2006). Transgenic worms expressing Aß_1-42_ in the muscles using the *myo-3* promoter show an accumulation of autophagosomes, and this phenotype was alleviated by *daf-2* mutations (Florez-McClure et al., 2007). In familial ALS models, crossing the *daf-2(e1370)* mutations into the transgenic worms expressing G85R (Boccitto et al., 2012) or G93A (Li et al., 2013) SOD1 mutations significantly ameliorated the motor deficits and the accumulation of insoluble aggregates. Likewise, transgenic worms overexpressing the full-length TDP-43 using a pan-neuronal *snb-1* promoter resulted in an accumulation of insoluble aggregates and neurotoxicity, whereas *daf-2* mutation reduced protein aggregates and alleviated disease phenotype (Zhang et al., 2011). Consistent with the reports in other neurodegenerative models, here we provide the evidence that the reduction of *daf-2* can improve the locomotion in *C. elegans* models of SMA. This observation is in line with work by Liu et al. (2013) showing that N2 worm locomotor function and neuromuscular transmission begin to decline in the second week of adulthood and this is blunted in the *daf-2* mutant background. The extent to which these observations in middle aged adult worms are mechanistically relevant to our results on the *smn-1* mutant animals will require further investigation.

### The *daf-2* pathway and locomotor defects in *smn-1* mutant animals

The locomotor function of animals lacking *smn-1* is comparable to wild type at early time points. However, normal developmental progression fails to occur in these animals and instead a severely uncoordinated phenotype emerges. The nervous system of *smn-1(ok355)* is, to a large degree, morphologically intact (Briese et al., 2009), although limited NMJ abnormalities do exist (Figs. 5, 6; O'Hern et al., 2017). A reasonable speculation for progressive motor deficits in *smn-1* mutant animals is circuit dysfunction, that is, diminished SMN in neurons that drive motor activation are impaired in their operation. This supposition is consistent with observations made in *Drosophila* (Imlach et al., 2012) and mouse (Mentis et al., 2011) models of SMA. In fly, the reduced SMN state leads to many defects in larval neuromuscular function and all of these abnormalities were rescued by pan neuronal transgenic expression of SMN. Interestingly, restitution of SMN in fly brain excitatory cholinergic neurons (but not motor neurons or muscle) also rescues these abnormalities. Similar observations have been made in the SMAΔ7 mouse models (McGovern et al., 2015); overexpression of SMN in motor neurons alone has a minimal effect on survival and the animals are weak, while overexpression of SMN in motor neurons and other neurons had a much more robust survival promoting effect. Further, elimination of SMN from motor neurons has no effect on survival or strength, while elimination of SMN from both motor neurons and other neurons leads to a shortened life span and weakness. Together, these data suggest that reduced SMN in neurons that drive motor neuron activation is a major determinant of abnormal motor function in models of SMA.

What is the nature of the defect incurred by reduced SMN in premotor interneurons that leads to abnormal neuromuscular unit function? In the fly model of SMA, distinct cholinergic neuron populations contribute to distinct aspects of motor function, and this led Imlach et al., to conclude that “…the effect of SMN depletion on the motor network is an amalgam of specific defects in distinct neurons that sum to produce a generalize disruption of the motor system…” our observations complement this formulation. We find that (1) reduction in *daf-2* signaling improves locomotion in the worm model of SMA, (2) this is associated with enhanced GABAergic neurotransmission, and (3) simply enhancing GABAergic transmission also improves locomotion. We propose that diminished GABAergic neurotransmission in the worm nervous system impairs the function of circuitry required for neuromuscular system operation in the *smn-1(ok355)* mutant animals. Consideration of the fly and worm data together supports the view that neurochemically defined populations of premotor interneurons are dysfunctional in the reduced SMN condition, and the sum of this interneuron dysfunction manifests as impaired motor behavior. It is noteworthy that the mouse (and likely human) spinal cord contains GABAergic and cholinergic subtypes of interneurons (Waldvogel et al., 1990; Caldeira et al., 2017). We suggest that manipulating the activity of subgroups of spinal cord interneurons could improve motor function in the reduced SMN condition.

### GABAergic neurotransmission in *C. elegans* models of SMA

*C. elegans* GABAergic neurotransmission is a fascinating topic. Classic work identifies 26 GABAergic neurons in the worm based on the criteria: (1) they contain GABA, (2) express the GABA biosynthetic enzyme glutamic acid decarboxylase (GAD, *unc-25*), and (3) express the GABA vesicular transporter (GAT, *unc-47*) for loading GABA into synaptic vesicles (Gendrel et al., 2016). Recent work suggests this is an underestimate and at least two other neuron types could be involved in neuronal GABAergic transmission. One class of neurons, containing GABA and GABA uptake transporter (*snf-11*), have been dubbed “GABA clearance neurons” and a second class of neurons, containing GABA, GABA uptake transporter (*snf-11*) and GABA vesicular transporter (*unc-47*), have been dubbed “GABA recycling neurons.” Based on their anatomy and position, both subtypes are poised to release GABA in addition to classical GABAergic neurons. Since 30–40% of all synapses in the worm have GABA receptors, GABA may have both widespread and precise influence on circuit behavior (Gendrel et al., 2016).

We find that two different ways of enhancing GABAergic transmission in the *smn-1(ok355)* mutant animals led to improved locomotion function, strong evidence that improvement in mechanical power of the SMA mutant animals is a result of increased GABAergic tone. There are 7 known GABA receptor subtypes in the worm (Jorgensen, 2005; Gendrel et al., 2016) and the UNC-49 locus encodes three splice variants; the *UNC-49::GFP* worms we used here is an integrated line with GFP inserted in-frame with the B splice variant (Bamber et al., 1999). Part of GABA receptor diversity is thought to arise from alternative splicing of the UNC-49 locus in a cell type-specific manner. The existence of multiple copies of the UNC-49 locus in the *UNC-49::GFP* worms we used is likely to result in increased GABA receptor occupancy at synapses. However, since SMN functions in spliceosome assembly, the *UNC-49::GFP;smn-1(ok355)* mutant animals may have a quantitatively and qualitatively unique GABA receptor phenotype. As mentioned above, the *snf-11* locus encodes the sole GABA uptake transporter in the worm and in the *snf-11(ok156)* mutant animals there is likely to be enhanced GABAergic neurotransmission owing to decreased GABA clearance. Considering the existence of GABA clearance and GABA recycling neurons, the *snf-11(ok156)* mutant animals may have a more widespread and perhaps less specific effect on interneuronal GABAergic communication. Although both mutant animals enhance the mechanical power of the *smn-1(ok355)* mutant animals, their different modes of action provide an explanation for why there is a restoration of body curvature when swimming in the *smn-1(ok355);UNC-49::GFP*, but not *smn-1(ok355);*s*nf-11(ok156)*, mutant animals.

### Perturbation of the balance of excitatory to inhibitory (E/I) neurotransmission in *C. elegans* models of SMA

Our anatomic (Figs. 5, 6), functional (Fig. 7), and genetic (Figs. 8, 9) studies are consistent with the view that an impairment of GABAergic neurotransmission is an important contributor to locomotor dysfunction in the *smn-1(ok355)* mutant animals. Studies of other neuronal populations come to complementary conclusions. For example, O'Hern et al. (2017) show that reduced levels of SMN leads to decreased microRNA-2 function, and this results in increased translation of GAR-2, the orthologue of the M2 muscarinic receptor (m2R). In mice, presynaptic M2R receptors control (i.e., inhibit) the time course of acetylcholine release from motor neurons (Slutsky et al., 2001; Parnas et al., 2005). The inference for the worm is that diminished SMN could enhance GAR-2 mediated inhibition of acetylcholine release and thereby impair the activation of cholinergic NMJs. However, since GAR-2 is expressed in a large number of neurons (Cao et al., 2017), increased GAR-2 expression is likely to be having a broad effect. Together with our observations, we suggest that an imbalance of E/I neurotransmission underlies, at least in part, the motor impairments in *smn-1(ok355)*. This raises the possibility that pharmacological manipulation of these systems could be of benefit to individuals with SMA

We note that the Hart lab reported *smn-1(ok355)* mutant animals to be resistant to aldicarb and levamisole (O'Hern et al., 2017), while we find *smn-1(ok355)* mutant animals switch from levamisole-resistant phenotype at L2 stage to both levamisole- and pyridostigmine-hypersensitive phenotype at the late stage. The origin of the disparity between these observations probably lies in differences in developmental stages, definition, and timing of paralyses, the Hart lab performed the functional tests at the L4 stage, while we performed the functional tests at the L2 and late (L2 + 3 d, the fifth day after egg drop) stage.

### Electrophysiological abnormalities in mouse models of SMA

Do observations in mouse models of SMA provide support for the view that anatomic/functional abnormalities in segmental spinal cord circuitry contribute to motor deficits? Study of the intrinsic properties of motor neurons and the monosynaptic reflex arc between proprioceptive afferents and motor neurons has revealed that SMAΔ7 motor neurons are hyperexcitable (Ling et al., 2010; Mentis et al., 2011). Despite this, stimulation of Ia afferents evokes smaller motor neuron potentials and this is likely to be due to either a reduced number of proprioceptive synapses on motor neurons, or a functional impairment of the remaining Ia-motor neuron synapses. This would be consistent with a specific reduction of VGlut1 expression on motor neuron soma in the SMAΔ7 mice (Ling et al., 2010; Mentis et al., 2011). There is only a small amount of data on inhibitory systems. McGovern et al. (2015) found that ablating SMN in GAD-expressing neurons had no phenotypic effects, and expressing SMN in GAD-expressing neurons in SMNΔ7 mice did not affect longevity. The extent to which these manipulations influence locomotion is unknown. Immunohistological studies reveal normal numbers of GABAergic (VGAT) and glycinergic (GlyT2) presynaptic terminals on motor neuron soma in the SMAΔ7 mice (Ling et al., 2010; Mentis et al., 2011). We do not know what effect reduced SMN has on the connectivity among segmental spinal cord interneurons, between segmental spinal cord interneurons and motor neurons, and the degree of functional alterations (i.e., patterns of activity) within these interneuronal populations. While technically challenging to study, we believe this is a critical gap in our understanding of why reduced SMN leads to weakness. Considering the remarkable molecular diversity of spinal cord interneurons (Bikoff et al., 2016), we predict selected subtypes of interneurons will be dysfunctional and/or aberrantly connected to partners in the reduced SMN state. These alterations could impair the activation of motor neurons leading to weakness.

## Synthesis

Reviewing Editor: Mike Fainzilber, Weizmann Institute of Science Decisions are customarily a result of the Reviewing Editor and the peer reviewers coming together and discussing their recommendations until a consensus is reached. When revisions are invited, a fact-based synthesis statement explaining their decision and outlining what is needed to prepare a revision will be listed below. The following reviewer(s) agreed to reveal their identity: NONE. Note: If this manuscript was transferred from JNeurosci and a decision was made to accept the manuscript without peer review, a brief statement to this effect will instead be what is listed below.

The manuscript by Wu and colleagues describes a new role of the insulin signalling pathway and GABAergic neurotransmission in a C. elegans model of SMA. Using loss of function mutants for smn-1 they are able to rescue several neuronal phenotypes by altering insulin signalling and/or GABA signalling.

The focus on GABA is interesting and novel and extend knowledge for the field that will most certainly be followed up in more advanced models. The daf-2 aspect of the work is less novel, but set the stage for the GABA discoveries (also, previous relevant daf-2 work is appropriately considered).

Please tone down this statement on page 7 “These effects are specific because reducing other genes previously implicated in lifespan extension did not extend lifespan in smn-1 null animals.” There are many genes implicated in lifespan, and here they tested a tiny fraction, and not even the main genes, so they cannot realistically conclude that this is ‘specific’.

There are a few typos, please proof read carefully before rersubmission: e.g. p. 19 “Simple observation of the swimming gait of individuals from each genotype over a one second demonstrates that smn-1...” or over a one second interval, or over one second.

Also please check abstract and significance statement carefully for typos and clarity.

## References

[B1] Arantes-Oliveira N, Apfeld J, Dillin A, Kenyon C (2002) Regulation of Life-Span by Germ-Line Stem Cells in Caenorhabditis elegans. Science 295:502–505. 10.1126/science.1065768 11799246

[B2] Bamber BA, Beg AA, Twyman RE, Jorgensen EM (1999) The *Caenorhabditis elegans* unc-49 locus encodes multiple subunits of a heteromultimeric GABA receptor. J Neurosci 19:5348–5359. 10377345 10.1523/JNEUROSCI.19-13-05348.1999 PMC6782323

[B3] Bikoff JB, Gabitto MI, Rivard AF, Drobac E, Machado TA, Miri A, Brenner-Morton S, Famojure E, Diaz C, Alvarez FJ, Mentis GZ, Jessell TM (2016) Spinal inhibitory interneuron diversity delineates variant motor microcircuits. Cell 165:207–219. 10.1016/j.cell.2016.01.027 26949184 PMC4808435

[B4] Biondi O, Branchu J, Ben Salah A, Houdebine L, Bertin L, Chali F, Desseille C, Weill L, Sanchez G, Lancelin C, Aïd S, Lopes P, Pariset C, Lécolle S, Côté J, Holzenberger M, Chanoine C, Massaad C, Charbonnier F (2015) IGF-1R Reduction Triggers Neuroprotective Signaling Pathways in Spinal Muscular Atrophy Mice. J Neurosci 35:12063–12079. 10.1523/jneurosci.0608-15.2015 26311784 PMC6705454

[B5] Boccitto M, Lamitina T, Kalb RG (2012) Daf-2 signaling modifies mutant SOD1 toxicity in *C. elegans*. PLoS One 7:e33494. 10.1371/journal.pone.0033494 22457769 PMC3308959

[B6] Bosch-Marce M, Wee CD, Martinez TL, Lipkes CE, Choe DW, Kong L, Van Meerbeke JP, Musaro A, Sumner CJ (2011) Increased IGF-1 in muscle modulates the phenotype of severe SMA mice. Human Molecular Genetics 20:1844–1853. 10.1093/hmg/ddr067 21325354 PMC3071675

[B7] Brenner S (1974) The genetics of *Caenorhabditis elegans* . Genetics 77:71–94. 4366476 10.1093/genetics/77.1.71 PMC1213120

[B8] Briese M, Esmaeili B, Fraboulet S, Burt EC, Christodoulou S, Towers PR, Davies KE, Sattelle DB (2009) Deletion of smn-1, the *Caenorhabditis elegans* ortholog of the spinal muscular atrophy gene, results in locomotor dysfunction and reduced lifespan. Hum Mol Genet 18:97–104. 10.1093/hmg/ddn320 18829666 PMC2644645

[B9] Burnett BG, Muñoz E, Tandon A, Kwon DY, Sumner CJ, Fischbeck KH (2009) Regulation of SMN protein stability. Mol Cell Biol 29:1107–1115. 10.1128/MCB.01262-08 19103745 PMC2643817

[B10] Caldeira V, Dougherty KJ, Borgius L, Kiehn O (2017) Spinal Hb9::Cre-derived excitatory interneurons contribute to rhythm generation in the mouse. Sci Rep 7:41369. 10.1038/srep41369 28128321 PMC5269678

[B11] Cao J, Packer JS, Ramani V, Cusanovich DA, Huynh C, Daza R, Qiu X, Lee C, Furlan SN, Steemers FJ, Adey A, Waterston RH, Trapnell C, Shendure J (2017) Comprehensive Single Cell Transcriptional Profiling of a Multicellular Organism by Combinatorial Indexing. bioRxiv. http://science.sciencemag.org/content/357/6352/661 28818938 10.1126/science.aam8940 PMC5894354

[B12] Chen ATY, Guo C, Itani OA, Budaitis BG, Williams TW, Hopkins CE, McEachin RC, Pande M, Grant AR, Yoshina S, Mitani S, Hu PJ (2015) Longevity genes revealed by integrative analysis of isoform-specific daf-16/FoxO mutants of *Caenorhabditis elegans* . Genetics 201:613–629. 10.1534/genetics.115.177998 26219299 PMC4596673

[B13] Cifuentes-Diaz C, Frugier T, Tiziano FD, Lacène E, Roblot N, Joshi V, Moreau MH, Melki J (2001) Deletion of murine SMN exon 7 directed to skeletal muscle leads to severe muscular dystrophy. J Cell Biol 152:1107–1114. 10.1083/jcb.152.5.1107 11238465 PMC2198815

[B14] Cohen E, Bieschke J, Perciavalle RM, Kelly JW, Dillin A (2006) Opposing activities protect against age-onset proteotoxicity. Science 313:1604–1610. 10.1126/science.1124646 16902091

[B15] Coque E, Raoul C, Bowerman M (2014) ROCK inhibition as a therapy for spinal muscular atrophy: understanding the repercussions on multiple cellular targets. Front Neurosci 8:271. 10.3389/fnins.2014.00271 25221469 PMC4148024

[B16] Dabbish NS, Raizen DM (2011) GABAergic Synaptic Plasticity during a Developmentally Regulated Sleep-Like State in C. elegans. J Neurosci 31:15932–15943. 10.1523/jneurosci.0742-11.2011 22049436 PMC3226813

[B17] Dillin A, Crawford DK, Kenyon C (2002) Timing requirements for insulin/IGF-1 signaling in *C. elegans* . Science 298:830–834. 10.1126/science.1074240 12399591

[B18] Dimitriadi M, Sleigh JN, Walker A, Chang HC, Sen A, Kalloo G, Harris J, Barsby T, Walsh MB, Satterlee JS, Li C, Van Vactor D, Artavanis-Tsakonas S, Hart AC (2010) Conserved genes act as modifiers of invertebrate SMN loss of function defects. PLoS Genet 6:e1001172 10.1371/journal.pgen.1001172 21124729 PMC2965752

[B19] Dimitriadi M, Derdowski A, Kalloo G, Maginnis MS, OHern P, Bliska B, Sorkaç A, Nguyen KCQ, Cook SJ, Poulogiannis G, Atwood WJ, Hall DH, Hart AC (2016) Decreased function of survival motor neuron protein impairs endocytic pathways. Proc Natl Acad Sci USA 113:E4377–E4386. 27402754 10.1073/pnas.1600015113 PMC4968725

[B20] Finkel RS, Chiriboga CA, Vajsar J, Day JW, Montes J, De Vivo DC, Yamashita M, Rigo F, Hung G, Schneider E, Norris DA, Xia S, Bennett CF, Bishop KM (2016) Treatment of infantile-onset spinal muscular atrophy with nusinersen: a phase 2, open-label, dose-escalation study. Lancet 388:3017–3026. 10.1016/s0140-6736(16)31408-8 27939059

[B21] Fischer U, Liu Q, Dreyfuss G (1997) The SMN-SIP1 complex has an essential role in spliceosomal snRNP biogenesis. Cell 90:1023–1029. 9323130 10.1016/s0092-8674(00)80368-2

[B22] Florez-McClure ML, Hohsfield LA, Fonte G, Bealor MT, Link CD (2007) Decreased insulin-receptor signaling promotes the autophagic degradation of beta-amyloid peptide in *C. elegans* . Autophagy 3:569–580. 10.4161/auto.4776 17675890

[B23] Fraser AG, Kamath RS, Zipperlen P, Martinez-Campos M, Sohrmann M, Ahringer J (2000) Functional genomic analysis of *C. elegans* chromosome I by systematic RNA interference. Nature 408:325–330. 10.1038/35042517 11099033

[B24] Gendrel M, Atlas EG, Hobert O (2016) A cellular and regulatory map of the GABAergic nervous system of *C. elegans* . Elife 5:1395. 10.7554/eLife.17686 PMC5065314 27740909

[B25] Gray J, Hancock GJ (1955) The propulsion of sea-urchin spermatozoa. J Exp Biol 32:802–814.

[B26] Gubitz AK, Feng W, Dreyfuss G (2004) The SMN complex. Exp Cell Res 296:51–56. 10.1016/j.yexcr.2004.03.022 15120993

[B27] Hamilton B, Dong Y, Shindo M, Liu W, Odell I, Ruvkun G, Lee SS (2005) A systematic RNAi screen for longevity genes in *C. elegans* . Genes Dev 19:1544–1555. 10.1101/gad.1308205 15998808 PMC1172061

[B28] Hansen M, Hsu A-L, Dillin A, Kenyon C (2005) New genes tied to endocrine, metabolic, and dietary regulation of lifespan from a *Caenorhabditis elegans* genomic RNAi screen. PLoS Genet 1:e17 10.1371/journal.pgen.0010017 PMC1183531 16103914

[B29] Henderson ST, Johnson TE (2001) daf-16 integrates developmental and environmental inputs to mediate aging in the nematode *Caenorhabditis elegans* . Curr Biol 11:1975–1980. 11747825 10.1016/s0960-9822(01)00594-2

[B30] Houthoofd K, Braeckman BP, Lenaerts I, Brys K, De Vreese A, Van Eygen S, Vanfleteren JR (2002) Axenic growth up-regulates mass-specific metabolic rate, stress resistance, and extends life span in Caenorhabditis elegans. Exp Gerontol 37:1371–1378. 10.1016/s0531-5565(02)00173-0 12559406

[B31] Hua Y, Sahashi K, Rigo F, Hung G, Horev G, Bennett CF, Krainer AR (2011) Peripheral SMN restoration is essential for long-term rescue of a severe spinal muscular atrophy mouse model. Nature 478:123–126. 10.1038/nature10485 21979052 PMC3191865

[B32] Hung W, Hwang C, Po MD, Zhen M (2007) Neuronal polarity is regulated by a direct interaction between a scaffolding protein, Neurabin, and a presynaptic SAD-1 kinase in *Caenorhabditis elegans* . Development 134:237–249. 10.1242/dev.02725 17151015

[B33] Ilieva HS, Yamanaka K, Malkmus S, Kakinohana O, Yaksh T, Marsala M, Cleveland DW (2008) Mutant dynein (Loa) triggers proprioceptive axon loss that extends survival only in the SOD1 ALS model with highest motor neuron death. Proc Natl Acad Sci USA 105:12599–12604. 10.1073/pnas.0805422105 18719118 PMC2527957

[B34] Imlach WL, Beck ES, Choi BJ, Lotti F, Pellizzoni L, McCabe BD (2012) SMN is required for sensory-motor circuit function in *Drosophila* . Cell 151:427–439. 10.1016/j.cell.2012.09.011 23063130 PMC3475188

[B35] Jia K, Chen D, Riddle DL (2004) The TOR pathway interacts with the insulin signaling pathway to regulate *C. elegans* larval development, metabolism and life span. Development 131:3897–3906. 10.1242/dev.01255 15253933

[B36] Jorgensen EM (2005) GABA (August 31, 2005), WormBook, ed. The C. elegans research community, WormBook.

[B37] Kariya S, Obis T, Garone C, Akay T, Sera F, Iwata S, Homma S, Monani UR (2014) Requirement of enhanced Survival Motoneuron protein imposed during neuromuscular junction maturation. J Clin Invest 124:785–800. 10.1172/jci72017 24463453 PMC3904626

[B38] Kenyon CJ (2010) The genetics of ageing. Nature 464:504–512. 10.1038/nature08980 20336132

[B39] Kenyon C, Chang J, Gensch E, Rudner A, Tabtiang R (1993) A *C. elegans* mutant that lives twice as long as wild type. Nature 366:461–464. 10.1038/366461a0 8247153

[B40] Kimura KD, Tissenbaum HA, Liu Y, Ruvkun G (1997) daf-2, an insulin receptor-like gene that regulates longevity and diapause in Caenorhabditis elegans. Science 277:942–946. 10.1126/science.277.5328.942 9252323

[B41] Kojima T, Kamei H, Aizu T, Arai Y, Takayama M, Nakazawa S, Ebihara Y, Inagaki H, Masui Y, Gondo Y, Sakaki Y, Hirose N (2004) Association analysis between longevity in the Japanese population and polymorphic variants of genes involved in insulin and insulin-like growth factor 1 signaling pathways. Exp Gerontol 39:1595–1598. 10.1016/j.exger.2004.05.007 15582274

[B42] Krajacic P, Shen X, Purohit PK, Arratia P, Lamitina T (2012) Biomechanical profiling of *Caenorhabditis elegans* motility. Genetics 191:1015–1021. 10.1534/genetics.112.141176 22554893 PMC3389964

[B43] Larsen PL, Albert PS, Riddle DL (1995) Genes that regulate both development and longevity in *Caenorhabditis elegans* . Genetics 139:1567–1583. 7789761 10.1093/genetics/139.4.1567 PMC1206485

[B44] Lauga E, Powers TR (2009) The hydrodynamics of swimming microorganisms. Rep Prog Phys 72:096601 10.1088/0034-4885/72/9/096601

[B45] Le TT, Pham LT, Butchbach MER, Zhang HL, Monani UR, Coovert DD, Gavrilina TO, Xing L, Bassell GJ, Burghes AHM (2005) SMNDelta7, the major product of the centromeric survival motor neuron (SMN2) gene, extends survival in mice with spinal muscular atrophy and associates with full-length SMN. Hum Mol Genet 14:845–857. 10.1093/hmg/ddi078 15703193

[B46] Lee RY, Hench J, Ruvkun G (2001) Regulation of *C. elegans* DAF-16 and its human ortholog FKHRL1 by the daf-2 insulin-like signaling pathway. Curr Biol 11:1950–1957. 11747821 10.1016/s0960-9822(01)00595-4

[B47] Lefebvre S, Burlet P, Liu Q, Bertrandy S, Clermont O, Munnich A, Dreyfuss G, Melki J (1997) Correlation between severity and SMN protein level in spinal muscular atrophy. Nat Genet 16:265–269. 10.1038/ng0797-265 9207792

[B48] Li J, Huang KX, Le WD (2013) Establishing a novel *C. elegans* model to investigate the role of autophagy in amyotrophic lateral sclerosis. Acta Pharmacol Sin 34:644–650. 10.1038/aps.2012.190 23503474 PMC3647213

[B49] Lighthill J (1976) Flagellar hydrodynamics. SIAM Rev 18:161–230. 10.1137/1018040

[B50] Lin K, Dorman JB, Rodan A, Kenyon C (1997) daf-16: an HNF-3/forkhead family member that can function to double the life-span of *Caenorhabditis elegans* . Science 278:1319–1322. 9360933 10.1126/science.278.5341.1319

[B51] Ling KKY, Lin MY, Zingg B, Feng Z, Ko CP (2010) Synaptic defects in the spinal and neuromuscular circuitry in a mouse model of spinal muscular atrophy. PLoS One 5:e15457 10.1371/journal.pone.0015457 21085654 PMC2978709

[B52] Liu J, Zhang B, Lei H, Feng Z, Liu J, Hsu AL, Xu XZS (2013) Functional aging in the nervous system contributes to age-dependent motor activity decline in *C. elegans* . Cell Metab 18:392–402. 10.1016/j.cmet.2013.08.007 24011074 PMC3811915

[B53] Mahoney TR, Luo S, Nonet ML (2006) Analysis of synaptic transmission in Caenorhabditis elegans using an aldicarb-sensitivity assay. Nat Protoc 1:1772–1777. 10.1038/nprot.2006.281 17487159

[B54] Martinez TL, Kong L, Wang X, Osborne MA, Crowder ME, Van Meerbeke JP, Xu X, Davis C, Wooley J, Goldhamer DJ, Lutz CM, Rich MM, Sumner CJ (2012) Survival motor neuron protein in motor neurons determines synaptic integrity in spinal muscular atrophy. J Neurosci 32:8703–8715. 10.1523/JNEUROSCI.0204-12.2012 22723710 PMC3462658

[B55] McGovern VL, Iyer CC, Arnold WD, Gombash SE, Zaworski PG, Blatnik AJ, Foust KD, Burghes AHM (2015) SMN expression is required in motor neurons to rescue electrophysiological deficits in the SMNΔ7 mouse model of SMA. Human Molecular Genetics 24:5524–5541. 10.1093/hmg/ddv283 26206889 PMC4572068

[B56] McWhorter ML, Monani UR, Burghes AHM, Beattie CE (2003) Knockdown of the survival motor neuron (Smn) protein in zebrafish causes defects in motor axon outgrowth and pathfinding. J Cell Biol 162:919–931. 10.1083/jcb.200303168 12952942 PMC1761110

[B57] Mentis GZ, Blivis D, Liu W, Drobac E, Crowder ME, Kong L, Alvarez FJ, Sumner CJ, O'Donovan MJ (2011) Early functional impairment of sensory-motor connectivity in a mouse model of spinal muscular atrophy. Neuron 69:453–467. 10.1016/j.neuron.2010.12.032 21315257 PMC3044334

[B58] Mercuri E, Darras BT, Chiriboga CA, Day JW, Campbell C, Connolly AM, Iannaccone ST, Kirschner J, Kuntz NL, Saito K, Shieh PB, Tulinius M, Mazzone ES, Montes J, Bishop KM, Yang Q, Foster R, Gheuens S, Bennett CF, Farwell W, et al. (2018) Nusinersen versus Sham Control in Later-Onset Spinal Muscular Atrophy. N Engl J Med 378:625–635. 29443664 10.1056/NEJMoa1710504

[B59] Monani UR (2005) Spinal muscular atrophy: a deficiency in a ubiquitous protein; a motor neuron-specific disease. Neuron 48:885–896. 10.1016/j.neuron.2005.12.001 16364894

[B60] Monani UR, Sendtner M, Coovert DD, Parsons DW, Andreassi C, Le TT, Jablonka S, Schrank B, Rossoll W, Rossol W, Prior TW, Morris GE, Burghes AH (2000) The human centromeric survival motor neuron gene (SMN2) rescues embryonic lethality in Smn(-/-) mice and results in a mouse with spinal muscular atrophy. Hum Mol Genet 9:333–339. 10.1093/hmg/9.3.333 10655541

[B61] Mullen GP, Mathews EA, Saxena P, Fields SD, McManus JR, Moulder G, Barstead RJ, Quick MW, Rand JB (2006) The *Caenorhabditis elegans* snf-11 gene encodes a sodium-dependent GABA transporter required for clearance of synaptic GABA. Mol Biol Cell 17:3021–3030. 10.1091/mbc.e06-02-0155 16641366 PMC1483038

[B62] O'Hern PJ, do Carmo G, Gonçalves I, Brecht J, López Soto EJ, Simon J, Chapkis N, Lipscombe D, Kye MJ, Hart AC (2017) Decreased microRNA levels lead to deleterious increases in neuronal M2 muscarinic receptors in spinal muscular atrophy models. Elife 6:e88427. 10.7554/eLife.20752 PMC5413352 28463115

[B63] Ogg S, Paradis S, Gottlieb S, Patterson GI, Lee L, Tissenbaum HA, Ruvkun G (1997) The fork head transcription factor DAF-16 transduces insulin-like metabolic and longevity signals in *C. elegans* . Nature 389:994–999. 10.1038/40194 9353126

[B64] Oh SW, Mukhopadhyay A, Dixit BL, Raha T, Green MR, Tissenbaum HA (2006) Identification of direct DAF-16 targets controlling longevity, metabolism and diapause by chromatin immunoprecipitation. Nat Genet 38:251–257. 10.1038/ng1723 16380712

[B65] Olsen A, Vantipalli MC, Lithgow GJ (2006) Checkpoint proteins control survival of the postmitotic cells in *Caenorhabditis elegans* . Science 312:1381–1385. 10.1126/science.1124981 16741121 PMC2568993

[B66] Oprea GE, Kröber S, McWhorter ML, Rossoll W, Müller S, Krawczak M, Bassell GJ, Beattie CE, Wirth B (2008) Plastin 3 is a protective modifier of autosomal recessive spinal muscular atrophy. Science 320:524–527. 10.1126/science.1155085 18440926 PMC4908855

[B67] Paez-Colasante X, Seaberg B, Martinez TL, Kong L, Sumner CJ, Rimer M (2013) Improvement of neuromuscular synaptic phenotypes without enhanced survival and motor function in severe spinal muscular atrophy mice selectively rescued in motor neurons. PLoS One 8:e75866 10.1371/journal.pone.0075866 24086650 PMC3781079

[B68] Parnas H, Slutsky I, Rashkovan G, Silman I, Wess J, Parnas I (2005) Depolarization initiates phasic acetylcholine release by relief of a tonic block imposed by presynaptic M2 muscarinic receptors. J Neurophysiol 93:3257–3269. 10.1152/jn.01131.2004 15703226

[B69] Rossoll W, Jablonka S, Andreassi C, Kröning A-K, Karle K, Monani UR, Sendtner M (2003) Smn, the spinal muscular atrophy-determining gene product, modulates axon growth and localization of beta-actin mRNA in growth cones of motoneurons. J Cell Biol 163:801–812. 10.1083/jcb.200304128 14623865 PMC2173668

[B70] Shababi M, Glascock J, Lorson CL (2011) Combination of SMN trans-splicing and a neurotrophic factor increases the life span and body mass in a severe model of spinal muscular atrophy. Hum Gene Ther 22:135–144. 10.1089/hum.2010.114 20804424

[B71] Shakkottai VG, do Carmo Costa M, Dell'Orco JM, Sankaranarayanan A, Wulff H, Paulson HL (2011) Early changes in cerebellar physiology accompany motor dysfunction in the polyglutamine disease spinocerebellar ataxia type 3. J Neurosci 31:13002–13014. 10.1523/JNEUROSCI.2789-11.2011 21900579 PMC3170039

[B72] Shen EZ, Song CQ, Lin Y, Zhang WH, Su PF, Liu WY, Zhang P, Xu J, Lin N, Zhan C, Wang X, Shyr Y, Cheng H, Dong MQ (2014) Mitoflash frequency in early adulthood predicts lifespan in *Caenorhabditis elegans* . Nature 508:128–132. 10.1038/nature13012 24522532

[B73] Sleigh JN, Buckingham SD, Esmaeili B, Viswanathan M, Cuppen E, Westlund BM, Sattelle DB (2011) A novel *Caenorhabditis elegans* allele, smn-1(cb131), mimicking a mild form of spinal muscular atrophy, provides a convenient drug screening platform highlighting new and pre-approved compounds. Hum Mol Genet 20:245–260. 10.1093/hmg/ddq459 20962036

[B74] Slutsky I, Silman I, Parnas I, Parnas H (2001) Presynaptic M(2) muscarinic receptors are involved in controlling the kinetics of ACh release at the frog neuromuscular junction. J Physiol 536:717–725. 11691867 10.1111/j.1469-7793.2001.00717.x PMC2278896

[B75] Suh Y, Atzmon G, Cho M-O, Hwang D, Liu B, Leahy DJ, Barzilai N, Cohen P (2008) Functionally significant insulin-like growth factor I receptor mutations in centenarians. Proc Natl Acad Sci USA 105:3438–3442. 10.1073/pnas.0705467105 18316725 PMC2265137

[B76] Sznitman J, Shen X, Sznitman R, Arratia PE (2010) Propulsive force measurements and flow behavior of undulatory swimmers at low Reynolds number. Phys Fluids 22:121901 10.1063/1.3529236

[B77] Waldvogel HJ, Faull RL, Jansen KL, Dragunow M, Richards JG, Mohler H, Streit P (1990) GABA, GABA receptors and benzodiazepine receptors in the human spinal cord: an autoradiographic and immunohistochemical study at the light and electron microscopic levels. Neuroscience 39:361–385. 1965016 10.1016/0306-4522(90)90274-8

[B78] Wolkow CA, Muñoz MJ, Riddle DL, Ruvkun G (2002) Insulin receptor substrate and p55 orthologous adaptor proteins function in the *Caenorhabditis elegans* daf-2/insulin-like signaling pathway. J Biol Chem 277:49591–49597. 10.1074/jbc.M207866200 12393910

[B79] Zhang T, Mullane PC, Periz G, Wang J (2011) TDP-43 neurotoxicity and protein aggregation modulated by heat shock factor and insulin/IGF-1 signaling. Hum Mol Genet 20:1952–1965. 10.1093/hmg/ddr076 21355045 PMC3080607

[B80] Zhang Z, Lotti F, Dittmar K, Younis I, Wan L, Kasim M, Dreyfuss G (2008) SMN deficiency causes tissue-specific perturbations in the repertoire of snRNAs and widespread defects in splicing. Cell 133:585–600. 10.1016/j.cell.2008.03.031 18485868 PMC2446403

[B81] Zhou C, Feng Z, Ko C-P (2016) Defects in motoneuron-astrocyte interactions in spinal muscular atrophy. J Neurosci 36:2543–2553. 10.1523/JNEUROSCI.3534-15.2016 26911699 PMC6705489

