# Supplementary figures and images for: Enhancing GABAergic Transmission Improves Locomotion in a Caenorhabditis elegans Model of Spinal Muscular Atrophy
Source: eNeuro. 2019 Jan 2;5(6):ENEURO.0289-18.2018. doi: 10.1523/ENEURO.0289-18.2018 (PMC6325564; doi:10.1523/ENEURO.0289-18.2018)

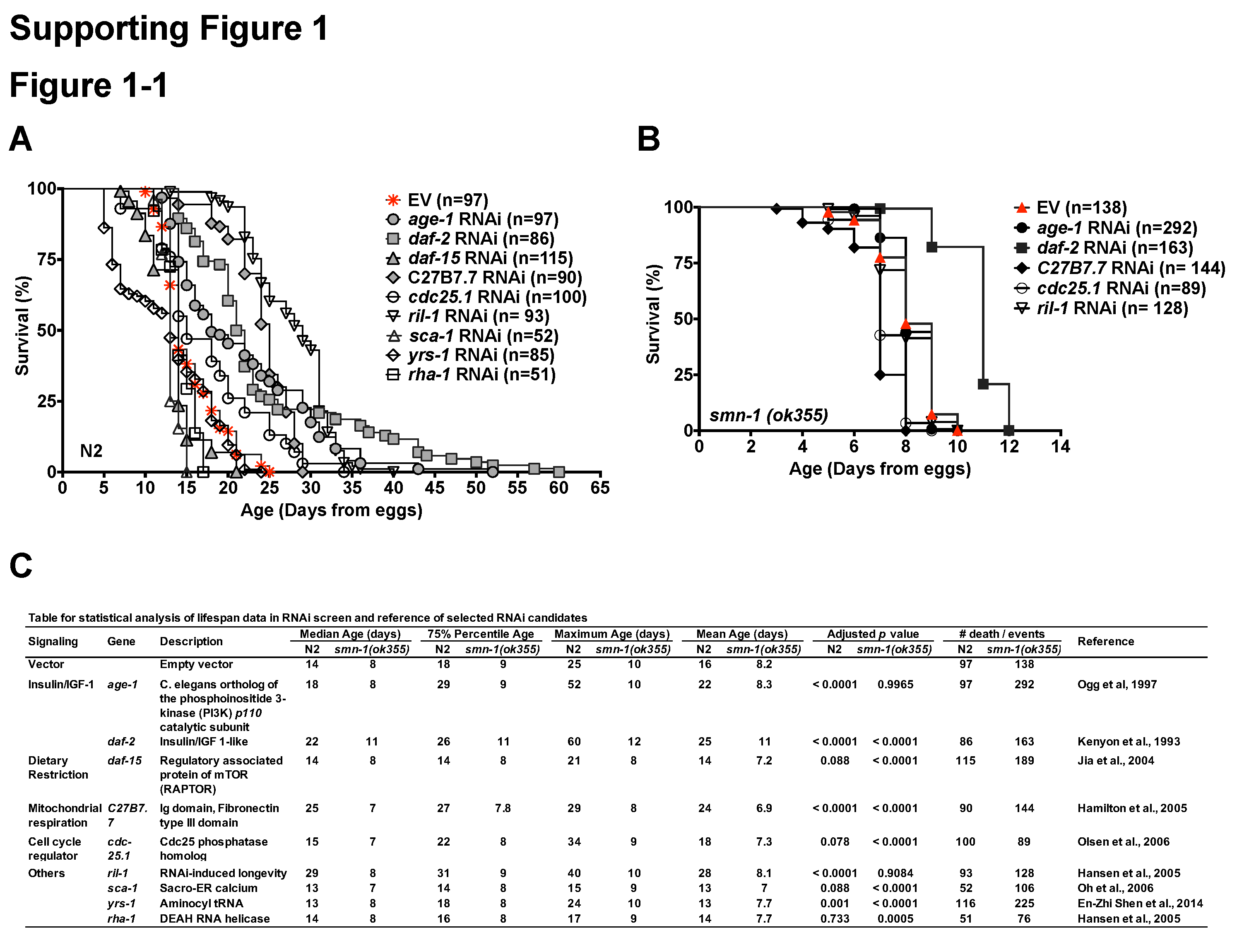

Supplement: Extended Data Figure 1-1 — An approach of RNAi screen identified Daf-2 as a disease modifier to promote lifespan in smn-1(ok355) mutant animals. A, Lifespan analysis of wild-type N2 worms fed either EV (median survival 14 d) or corresponding RNAi clones, including age-1 RNAi (median survival 18 d), daf-2 RNAi (median survival 21.5 d, p < 0.001, Log-rank test), daf-15 RNAi (median survival 14 d), C27B7.7 RNAi (median survival 25 d, p < 0.001, Log-rank test), cdc25 RNAi (median survival 15 d, p < 0.001, Log-rank test), ril-1 RNAi (median survival 29 d, p < 0.001, Log-rank test), sca-1 RNAi (median survival 13 d), yrs-1 RNAi (median survival 13 d), or rha-1 RNAi (median survival 14 d); p < 0.005 was considered significant after Bonferroni correction. B, Lifespan analysis of smn-1(ok355) mutant animals fed either EV (median survival 8 d) or longevity-promoting RNAi clones validated in Figure 1A. Only daf-2 RNAi feeding significantly prolongs lifespan in smn-1(ok355) mutant animals (median survival 11 d, p < 0.0001, Log-rank test); p < 0.006 was considered significant after Bonferroni correction. C, Table for statistical analysis of lifespan data in RNAi screen and reference of selected RNAi candidates. After conducting a literature search, nine candidate RNAi clones, which can extend lifespan in C. elegans, were selected for RNAi screen experiment. Download Figure 1-1, TIF file. [file sup_enu-eN-NWR-0289-18-s01.tif]
